# Mesoscale eddies shape *Prochlorococcus* community structure and dynamics in the oligotrophic open ocean

**DOI:** 10.1093/ismejo/wraf106

**Published:** 2025-05-24

**Authors:** Uri Sheyn, Kirsten E Poff, John M Eppley, Andy O Leu, Jessica A Bryant, Fuyan Li, Anna E Romano, Andrew Burger, Benedetto Barone, Edward F DeLong

**Affiliations:** Daniel K. Inouye Center for Microbial Oceanography: Research and Education, Department of Oceanography, University of Hawai’i, Honolulu, HI 96822, United States; Department of Biological Sciences, Virginia Tech, Blacksburg, VA 24061, United States; Daniel K. Inouye Center for Microbial Oceanography: Research and Education, Department of Oceanography, University of Hawai’i, Honolulu, HI 96822, United States; Daniel K. Inouye Center for Microbial Oceanography: Research and Education, Department of Oceanography, University of Hawai’i, Honolulu, HI 96822, United States; Daniel K. Inouye Center for Microbial Oceanography: Research and Education, Department of Oceanography, University of Hawai’i, Honolulu, HI 96822, United States; Centre for Microbiome Research, School of Biomedical Sciences, Queensland University of Technology (QUT), Translational Research Institute, Woolloongabba, QLD 4102, Australia; Daniel K. Inouye Center for Microbial Oceanography: Research and Education, Department of Oceanography, University of Hawai’i, Honolulu, HI 96822, United States; Seres Therapeutics, Inc., Cambridge, MA 02140, United States; Daniel K. Inouye Center for Microbial Oceanography: Research and Education, Department of Oceanography, University of Hawai’i, Honolulu, HI 96822, United States; Daniel K. Inouye Center for Microbial Oceanography: Research and Education, Department of Oceanography, University of Hawai’i, Honolulu, HI 96822, United States; Division of Chemistry and Chemical Engineering, California Institute of Technology, Pasadena, CA 91125, United States; Daniel K. Inouye Center for Microbial Oceanography: Research and Education, Department of Oceanography, University of Hawai’i, Honolulu, HI 96822, United States; Daniel K. Inouye Center for Microbial Oceanography: Research and Education, Department of Oceanography, University of Hawai’i, Honolulu, HI 96822, United States; Daniel K. Inouye Center for Microbial Oceanography: Research and Education, Department of Oceanography, University of Hawai’i, Honolulu, HI 96822, United States

**Keywords:** *Prochlorococcus* ecotypes, mesoscale ocean eddies, deep chlorophyll maximum, nitrogen acquisition, North Pacific Subtropical Gyre, microbial community structure, oligotrophic ocean environments, population dynamics

## Abstract

Mesoscale eddies, horizontally rotating currents sometimes referred to as “ocean weather,” influence open ocean macronutrient distributions, primary production, and microbial community structure. Such eddies impact ecosystems like the North Pacific Subtropical Gyre, where year-round thermal stratification limits the mixing of subsurface macronutrients with surface waters. Populations of the dominant primary producer *Prochlorococcus* in the North Pacific Subtropical Gyre consist of genetic variants with differential adaptive traits to light intensity, temperature, and macronutrient availability. How *Prochlorococcus* population variants respond to transient, localized environmental changes, however, remains an open question. Leveraging microbial community phylogenetic, metagenomic, and metatranscriptomic data, we report here a consistent, specific enrichment of *Prochlorococcus* high-light I ecotypes around the deep chlorophyll maximum (DCM) in cyclonic eddies, but not adjacent anticyclonic eddies. The shallower DCM depths of cyclones had lower temperatures, higher light intensities, and elevated nutrient concentrations compared to adjacent anticyclones, which favored *Prochlorococcus* high-light I ecotype proliferation. *Prochlorococcus* high-light I ecotypes in the cyclone DCM exhibited unique genetic traits related to nitrogen metabolism and were enriched in gene transcripts associated with energy production, cell replication, and proliferation. *Prochlorococcus* gene transcripts involved in amino acid transport, metabolism, and biosynthesis were also elevated in the cyclone. These results suggest the potential importance of nitrogen metabolism in *Prochlorococcus* high-light I ecotype proliferation in cyclonic eddies. Our findings demonstrate how mesoscale eddies shape microbial community structure in the oligotrophic ocean and how *Prochlorococcus* communities respond to short-term localized environmental variability.

## Introduction

In the oligotrophic open ocean of the North Pacific Subtropical Gyre (NPSG) [1–3] microbial communities exist in a thermally stratified water column year-round. The relatively shallow surface mixed-layer depth, ranging from extremes of 20 m to 120 m [4, 5], has an average of 30–40 m in summer and fall, and 70–90 m in the winter, which limits exchange between surface and deeper, nutrient-rich waters [6–8]. Consequently, the photic zone of the NPSG can be represented as two distinct layers: a more nutrient-limited upper layer with low new production and a subsurface layer with higher new productivity and export of organic matter [9–12]. Here, in surface waters, summer blooms of nitrogen-fixing diazotrophs can provide nitrogenous nutrients that help support episodic new primary production and organic matter export to the deep sea [7, 13]. In contrast, new production in the subsurface deep chlorophyll maximum (DCM) layer (the deepest zone where photosynthesis still sustains net positive primary production) relies primarily on diffusion from the nutrient-rich waters beneath the photic zone [6, 14].

Within this persistently stratified environment of the NPSG, mesoscale eddies (horizontally rotating currents ~100 kilometers in width) can introduce dynamic physical variability that has the potential to redistribute nutrients, enhance primary production, and shape microbial community structure and function [15–26]. Unlike upwelling and mixing events, cyclonic eddies (counterclockwise horizontally rotating currents having negative sea-level anomalies (SLA)) can bring nutrient-rich deep water closer to the euphotic zone along with uplifted isopycnals (density layers), a process known as “eddy pumping”. This may enhance primary productivity in the lower photic zone without perturbing the persistent water column stratification. In contrast to cyclones, anticyclones (clockwise rotating horizontal currents having positive SLAs) exhibit the opposite effect, effectively pushing density layers of water downward from the surface [7, 13, 20, 21, 24, 25, 27–30].

With respect to the biological impacts of eddy dynamics in ocean systems, past work has demonstrated elevated biomass accumulation in the DCM of cyclonic eddies [20, 27, 30, 31]. In contrast, anticyclonic eddies are associated with a deeper DCM layer where photoautotrophs exhibit photoacclimation due to reduced light availability. Previous studies have demonstrated the potential of cyclones in NPSG to contribute to new productivity [20, 27, 30, 31], and an increased eukaryotic phytoplankton abundance, which has also been reported in the DCM of cyclones [20].

Although previous studies have documented environmental differences at the base of the photic zone between cyclonic and anticyclonic eddies [14, 16, 20, 24, 25, 27, 30, 32–34], it remains unclear how these perturbations affect microbial community structure and how those community changes, in turn, impact biogeochemical processes. Both heterotrophic bacteria and abundant photoautotrophs like *Prochlorococcus,* could be influenced by eddy-induced changes in light [35–37], oxygen [37, 38], temperature [39], inorganic nutrients (nitrate, nitrite, ammonia [40], phosphate [41], and iron [24]), and organic nutrients (urea [42], amino acids [43, 44]), especially at the DCM.

*Prochlorococcus* is the predominant photoautotroph in the NPSG, and its naturally occurring populations are comprised of genetically diverse variants that can be generally divided into high light (HL) and low light (LL) clades (herein referred to as “ecotypes”). Ecotype genetic variants exhibit differences in water column distributions, genome size and gene content, GC content, growth temperature optima, nutrient acquisition strategies, and other adaptive traits [35, 45–49]. To date, only minor seasonal fluctuations in *Prochlorococcus* populations have been observed in time-series studies in the NPSG, with consistent ecotype-specific abundance maxima occurring at distinct depths: the HLII ecotype dominates the surface, whereas various LL ecotypes (LLI, LLII, LLIII, and LLIV) prevail in the deeper photic zone, including the DCM [47]. Prior studies have shown that short-term environmental changes (hours to days) appear insufficient to cause shifts in *Prochlorococcus* ecotype community structure [50]. Instead, high-light-adapted ecotypes appear to undergo adaptive photoacclimation over these short intervals of environmental perturbation without exhibiting dramatic changes in their overall community structure [50, 51].

The central question we addressed in this work is: Can short-term, ephemeral environmental changes across relatively small spatial scales in the NPSG (such as those caused by mesoscale eddies) influence microbial succession and community structure in an otherwise stable, stratified ecosystem? Specifically, we investigated the influence of open-ocean mesoscale eddies on microbial community structure and function, with a focus on the dominant primary producer in this habitat, *Prochlorococcus*. We collected and analyzed samples from two different eddy-focused expeditions that transected adjacent cyclone-anticyclone pairs during May 2016 (HOE-Legacy 4 expedition) and May–June 2017 (MESO-SCOPE expedition) in the NPSG [25]. Using 16S ribosomal RNA gene amplicons, internal transcribed spacer (ITS) sequences, metagenomic, and metatranscriptomic surveys, we characterized the community structure of *Prochlorococcus* in adjacent cyclonic and anticyclonic eddies. We also leveraged metagenomic and metatranscriptomic gene-centric datasets to identify specific functions differentiating cyclonic versus anticyclonic *Prochlorococcus* communities. Our combined observations and analyses support the hypothesis that transient, localized eddy-induced environmental perturbations impact microbial community structure and function in open ocean ecosystems.

## Materials and methods

### Eddy sample collections

Samples were collected from pairs of adjacent eddies of opposite polarity during two oceanographic cruises: the spring 2016 HOE-Legacy 4 cruise (KOK1607; May 9–May 14, 2016) and the summer 2017 MESO-SCOPE cruise (KM1709; June 26–July 15, 2017). High-resolution depth profiles of seawater samples were collected from 15 depths at 5-meter intervals around the DCM at three main sampling stations in each eddy pair (in the anticyclonic and cyclonic eddy centers and at the front separating the pairs). For the 2016 HOE-Legacy 4 cruise, high-resolution sampling for the cyclone center occurred on May 12, 2016, and for the corresponding anticyclone center on May 11, 2016. For the 2017 MESO-SCOPE cruise, high-resolution sampling for the cyclone center occurred on July 3, 2017, and for the corresponding anticyclone center on July 8, 2017.

### MESO-SCOPE Lagrangian station sampling

Metatranscriptomic samples were collected from a depth of 15 m and from a deeper isopycnal surface that tracked the displacement of ecosystems near the DCM in each eddy. Sampling was conducted at ~4-hour intervals over three days during Lagrangian experiments near the center of cyclonic and anticyclonic eddies (n = 18 per eddy) as part of the MESO-SCOPE cruise. Lagrangian sampling followed a surface velocity program (SVP) drifter, as described before [25]. The deeper isopycnal surface to be sampled was determined based on the potential density at the chlorophyll maximum observed during pre-diel sampling casts [25]. The isopycnal density for the DCM was σθ = 25.24 kg m^−3^.

### Seawater collection and DNA/RNA sample processing

Seawater was collected using Niskin sampling bottles attached to the CTD rosette. For DNA/RNA processing, 1–2 L of seawater was filtered onto 0.2 μm Supor Membrane Disc filters (Pall, Port Washington, NY, USA) housed in Swinnex units (MilliporeSigma, Burlington, MA, USA) or polypropylene filter holders (Cole-Parmer, Vernon Hills, IL, USA; EW-06623-32) using a peristaltic pump at a flow rate of ~6 L/h. Total filtration times for metatranscriptomic samples ranged from 15 to 20 minutes. After filtration, the >0.2 μm cell-enriched samples were placed in RNALater (Thermo Fisher Scientific, Waltham, MA, USA; AM7021) and stored at −80°C until further processing.

### DNA processing for sequencing

DNA extraction was performed using a previously described procedure [52]. Briefly, particles were lysed on the filters, and DNA was purified from the lysate on Chemagen’s Magnetic Separation Module instrument (PerkinElmer, Waltham, MA, USA) using Chemagen DNA Saliva buffer kit (PerkinElmer, Waltham, MA, USA; CMG-1037-1). DNA was sheared using microTUBE-15 AFA Beads (Covaris, Woburn, MA, USA; part no. 520145). Fragment sizes were quantified using a Fragment Analyzer system (Agilent Technologies, Santa Clara, CA, USA; DNF-488-0500), and shear times on a Covaris M220 instrument (Covaris, Woburn, MA, USA) were customized to yield ~350 bp fragments. Libraries were prepared using TruSeq Nano LT kits (Illumina, San Diego, CA, USA, NP-101-1001) for 350 bp insert size. Libraries were quantified using PicoGreen (Thermo Fisher Scientific, Waltham, MA, USA; P11496) and normalized to equal concentrations before pooling.

### Amplicon sequencing and processing

Prokaryotic rRNA gene amplicon libraries were produced by PCR amplification of the V4 region of the 16S rRNA gene using barcoded 515F (5′GTGYCAGCMGCCGCGGTAA3′) and 806R (5′GGACTACNVGGGTWTCTAAT3′) primers, including the modifications introduced in subsequent studies [53–55]. Pooled libraries were sequenced on a MiSeq system using the MiSeq Reagent Kits v2 300-cycle chemistry (Illumina, San Diego, CA, USA; MS-102-2002). Sequences were demultiplexed with QIIME2 [56]. Error modeling and correction were done using DADA2 [57]. We identified *Prochlorococcus*, *Synechococcus*, and other cyanobacteria by assigning taxonomic annotations (Kingdom to Genus levels) to ASVs using the SILVA v132 database, and confirmed these assignments with the updated SILVA v138.1 database [58]. *Prochlorococcus* ecotype classifications were determined through reference sequence comparison and phylogenetic analysis (see Supplementary Methods for details). All taxonomic and ecotype annotations were further validated using the ProPortal-ASV-Annotation tool [59]. All sequencing reads are available from NCBI: HOE-Legacy 4 cruise bacterial 16S rRNA gene amplicons are available at https://www.ncbi.nlm.nih.gov/bioproject/707586. MESO-SCOPE cruise bacterial 16S rRNA gene amplicons are available at https://www.ncbi.nlm.nih.gov/bioproject/PRJNA596510.

### Metagenomic data generation and read mapping

Metagenomic libraries were generated from DNA extracted from filtered water samples collected during MESO-SCOPE cruise high-resolution vertical sampling in cyclonic and anticyclonic eddies, as described above. Libraries were prepared using the TruSeq Nano DNA library preparation kit (Illumina, San Diego, CA, USA; FC-121-4001) and sequenced using a 150 bp paired-end NextSeq500/550 High Output v2 reagent kit (Illumina, San Diego, CA, USA; FC-404-2002), with 1% PhiX (Illumina, San Diego, CA, USA; FC-110-3001) added for quality control. DNA sequence reads were demultiplexed using Illumina’s Bcl2fastq utility. Reads were verified and screened for quality using BBMap [60] (v38.90, www.sourceforge.net/projects/bbmap/) to remove adapters and phiX, BFC [61] (r181) to correct sequencing errors, and using Trimmomatic [62] (v0.39) to remove low-quality bases. Quality-controlled reads, averaging 9–10 million per sample, were then assembled, sample by sample, using megahit [63] (v1.2.9). Water column profile metagenomic sequences from the MESO-SCOPE cruise are available at https://www.ncbi.nlm.nih.gov/bioproject/PRJNA596510.

For metagenomic read mapping, the reads were aligned against a database of known *Prochlorococcus* ecotypes using Burrows-Wheeler alignment (BWA-MEM [64]; v0.7.17) with a 75-bp length cut-off corresponding to ~100% identity. *Prochlorococcus*-specific genes were identified using the Genome Taxonomy Database [65] with a 100% average nucleotide identity (ANI) cut-off and annotated using the Kyoto Encyclopedia of Genes and Genomes (KEGG) [66]. Genes were aggregated by KEGG Orthology (KO) to describe their molecular functions. The KOs were then correlated with the average read numbers per billion for HLI and HLII-specific sequences from a known *Prochlorococcus* ecotype database. To identify genes over-represented in cyclones, normalized reads from cyclone samples at or above the DCM were compared with those from anticyclone samples. Differences were calculated using both a difference/total ratio and log ratios, forming a list of genes enriched in the cyclone and significantly correlated with known HLI *Prochlorococcus* sequences.

### Functional trait enrichment analysis in *Prochlorococcus* HLI ecotypes

To identify *Prochlorococcus* HLI-specific functional traits, Pearson correlations were calculated between the reads per billion (RPB) of different *Prochlorococcus* genes (aggregated by KO annotations) and the average RPB of *Prochlorococcus* HLI or HLII sequences in the MESO-SCOPE metagenomic dataset. KO enrichment between eddies was determined by computing the difference in RPB values between cyclones and anticyclones, normalized by the total RPB sum for each KO (difference/total ratio). Genes meeting the following criteria were selected for further analysis: Significant Pearson correlations (*P* values <0.01) with *Prochlorococcus* HLI ecotypes, Correlation coefficients, and difference/total ratios >0.5. Functional annotation of enriched genes was performed using KEGG and eggNOG databases to classify metabolic pathways and cellular processes.

### RNA samples processing and metatranscriptomic sequencing

RNA extractions were performed by first removing RNALater (via centrifugation and pipetting), adding 300 μl of Ambion denaturing solution (Ambion, Austin, TX) directly onto the filter, spiking in External RNA Controls Consortium (ERCC) ExFold RNA Spike-In Mixes (Mix #1; Thermo Fisher Scientific, Waltham, MA, USA; 4 456 739), and vortexing for 1 min. Nuclease-free water (750 μl) was added to the sample, and the samples were purified and DNase-treated using a Chemagen MSM I instrument with the tissue RNA CMG-1212A kit (PerkinElmer, Waltham, MA, USA; CMG-1212A). Samples were enriched for mRNA by removal of rRNA using Ribozero (Illumina, San Diego, CA, USA; MRZB12424). The quality of purified RNA was assessed on a Fragment Analyzer system using the High Sensitivity RNA Analysis Kit (Agilent Technologies, Santa Clara, CA, USA; DNF-472-0500) and quantified using the RiboGreen RNA Assay Kit (Thermo Fisher Scientific, Waltham, MA, USA; R11491). cDNA was synthesized, and sequencing libraries were prepared using the ScriptSeq v2 RNA-Seq Library Preparation Kit (Illumina, San Diego, CA, USA; SSV21124). Unique single-plex barcodes were annealed onto cDNA fragments during the PCR enrichment for Illumina sequencing primers over 12 cycles, following the manufacturer’s guidelines. Libraries were normalized to a final DNA concentration of 4 nM, pooled in equimolar volumes, and sequenced on an Illumina NextSeq 500 system using the NextSeq 500 High Output Kit v2 (300-cycle; Illumina, San Diego, CA, USA; catalog no. FC-404-2002), with PhiX Control spiked in at ~5% for quality control. A total of 2 million, 150 bp paired-end reads were produced for each sample. Sequence files are available in the NCBI SRA archive at: https://www.ncbi.nlm.nih.gov/bioproject/PRJNA596510.

### Metatranscriptomic read processing and mapping

Sequence reads were verified and screened for quality using BBMap [60] (v38.73, www.sourceforge.net/projects/bbmap/) to remove adapters and phiX, BFC [61] (r181) to correct sequencing errors, and Trimmomatic [62] (v0.39) to remove low-quality bases. Raw sequence reads were submitted to the NCBI SRA under project number PRJNA596510. Cleaned reads were assembled using RNA-SPAdes [67] (v3.13.2), and genes were predicted from assembled transcripts using Prodigal [68] (v2.6.3). CD-HIT [69] (v4.8.1) was used to dereplicate transcript sequences at 97% amino acid identity cutoff using default settings and to merge the resulting catalog with genes from the ALOHA 2.0 dataset [52]. The resulting catalog of genes was annotated as follows. Putative taxonomic assignments were made based on best hits to genes from the GTDB [70] (r95; for Bacteria and Archaea) and PhyloDB [71] (r1.076; for Eukaryotes and Viruses) gene databases, using lastal [72] homology searches (v1060; with params: -F 5 -b 1 -x 15 -y 7 -z 25). KEGG orthologs were assigned using lastal homology (same as above) to the KEGG genes database [66] downloaded on 16/11/2020. EggNOG 5.0 families were assigned using eggNOG-mapper v2.0.1 [73]. Transcripts were identified and counted by mapping cleaned reads against this combined gene catalog using BWA-MEM [64] (v0.7.17).

Transcript counts were normalized to internally added ERCC standards to account for methodological biases between samples, including library preparation and sequencing. Transcript counts were also normalized to the volume of water filtered for each sample, producing transcript values per ml. To use the ERCC spike-in as an internal standard in each sample, the correction factor was calculated as follows: for each ERCC standard, known quantities of molecules that were spiked-in were compared to the counts retrieved from sequenced samples by read mapping with BBMap [60] (v38.73). The correction factor was calculated as the slope of the best-fit line going through these pairs of values, with an intersection forced to the origin. The detection limit corresponded to the lowest molar amount of the ERCC sequence detected in each sample. The species-level/genus (using GTDB taxonomy annotation [70]) transcript sum in each sample was used to calculate the representation of each species-level/genus between the eddies at the DCM depth and identify overrepresented species-level/genus according to their average sum fold change (anticyclone/cyclone). To calculate the fold-change between the eddies for each species-level/genus in the DCM, we divided the species-level/genus sum average of the anticyclone samples (n = 18) by the species-level/genus sum average of the cyclone sample (n = 18). *P* values for each species-level/genus were calculated using the Kruskal-Wallis H-test, implemented in the Python scipy package [74] (v1.3.1), and corrected for false detection rate using the Benjamini-Hochberg procedure implemented in the Python statsmodels package [75] (v 0.10.1). Transcript count values above detection limits were further normalized to each species-level annotation's total sample expression sum. This step was needed to compare eddy differential gene expression based on cellular regulation rather than the total number of cells. PyDESeq2 (Python implementation of DESeq2, v 0.4.0) was used to compare the species-level-specific transcripts expression between the eddies and identify overexpressed transcripts using the default Wald test and Benjamini-Hochberg procedure for false detection rate and *P* values adjustment [76].

## Results and discussion

### *Prochlorococcus* community structure varies between cyclonic versus anticyclonic eddies

**Figure 1:**
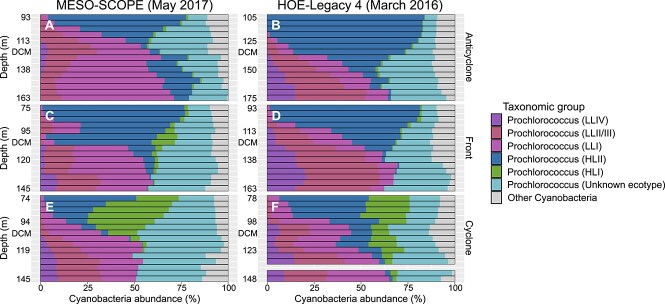
Differential representation of *Prochlorococcus* amplicon sequence variants (ASVs) in cyclones and anticyclones. Depth profiles of relative abundances of *Prochlorococcus* ASVs, centered around the DCM in 5 m depth increments, across mesoscale oceanographic features: Anticyclones (**A, B**), fronts (**C, D**), and cyclones (**E, F**). Data are shown for the MESO-SCOPE (**A, C, E**) and HOE-legacy 4 (**B, D, F**) research expeditions. ASVs are colored according to their corresponding 16S rRNA gene ecotype affiliations, highlighting consistent and distinct patterns of *Prochlorococcus* ecotype distribution between cyclonic and anticyclonic centers.

Depth-resolved 16S rRNA gene amplicon profiles (Supplementary Table S1) revealed a pronounced enrichment of *Prochlorococcus* HLI (ASV16) above the DCM in cyclonic eddies relative to anticyclonic eddies and the inter-eddy frontal region during both the MESO-SCOPE and HOE-Legacy 4 cruises (Fig. 1). This pattern was statistically significant across both expeditions (*P*-value = 0.0006 and 0.0002, for HOE-Legacy 4 and MESO-SCOPE, respectively; Kruskal-Wallis rank sum test; Supplementary Table S2), indicating a consistent ecotype shift associated with cyclonic eddy environments.

To validate ecotype assignments of ASVs, we compared them to full-length 16S rRNA gene sequences from the ProPortal *Prochlorococcus* genome and GTDB databases [59, 77] and conducted phylogenetic analyses using full-length 16S-ITS-23S rRNA genes clone libraries generated from DCM samples collected during MESO-SCOPE. These analyses confirmed the placement of ASV16 within the HLI ecotype (Supplementary Fig. 1; Supplementary Table S3). Additional ITS-based phylogenies further corroborated these assignments, establishing a direct correspondence between ASVs and ITS-defined ecotypes [78] (Supplementary Fig. 2, refer to the Methods section and Supplementary Methods for a comprehensive description of sequencing, annotation, and phylogenetic methods). These results highlight a consistent ecotype-specific response to mesoscale physical forcing, with HLI variants preferentially occupying the shoaled DCM of cyclonic eddies.

**Figure 2:**
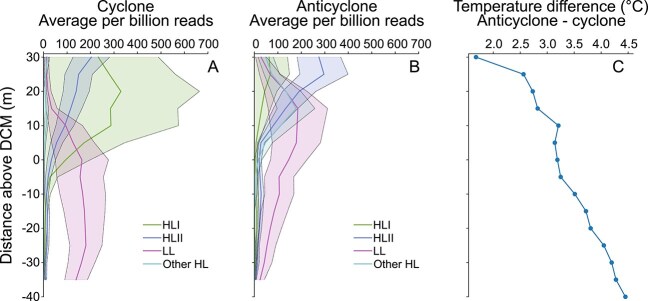
*Prochlorococcus* genetic variants are differentially represented in open ocean cyclones versus anticyclones. Internally transcribed spacer (ITS) sequences from known and cultivated *Prochlorococcus* isolates were mapped against metagenomes using the Burrows-Wheeler Aligner and manual curation. Mean mapped reads per billion sampled for *Prochlorococcus* ecotypes are displayed with 95% confidence intervals (shaded regions) for the cyclone (**A**) and anticyclone (**B**). Temperature differences between the anticyclone and the cyclone at depth above the DCM (**C**).

To confirm the enrichment of the *Prochlorococcus* HLI ecotype in cyclones (Fig. 1), we analyzed picoplankton metagenomic samples collected at the center of each eddy during the MESO-SCOPE cruise. These analyses revealed that the *Prochlorococcus* community in the cyclone was enriched in ITS sequences of *Prochlorococcus* strains MED4 and EQPAC1, which are both HLI ecotypes [79] (Fig. 2A). In contrast, HLII and LL ecotype ITS sequences dominated at all depths in the adjacent anticyclone (Fig. 2B). The enrichment of HLI ecotype ITS sequences just above the DCM of the cyclone but not in the anticyclone (Fig. 2A, B) is consistent with our 16S rRNA gene ASV results (Fig. 1). These results are also consistent with previous work showing that *Prochlorococcus* HLII ecotypes are typically the most highly represented (often by several orders of magnitude compared to HLI ecotypes) in the upper water column in lower latitudes of the NPSG near our study site [47, 50, 80].

A similar pattern of HLI enrichment in the cyclone was observed in a parallel metatranscriptomic analysis of DCM samples collected during the MESO-SCOPE cruise diel study. Biomass was sampled every 4 hours over 72 hours (n = 18) at the DCM depth from the centers of both eddies (Supplementary Fig. 3). To capture finer taxonomic resolution in our transcriptomic data, we used the Genome Taxonomy Database (GTDB) [81] to annotate *Prochlorococcus* transcripts at the genus and species levels. By extracting ITS sequences from GTDB genomes via *in silico* PCR (see Supplementary Methods section), we assign ecotype annotations to each GTDB species (Supplementary Table S4). Species within the GTDB genera of *Prochlorococcus A* included only genomes classified as HL ecotypes based on ITS sequences (Supplementary Fig. 4). According to the HLI cyclone enrichment trend we observed in the 16S rRNA gene ASV and metagenomic data (Fig. 1 and Fig. 2), we focused our transcriptomics analysis on transcripts belonging to *Prochlorococcus A* (all HL ecotypes).

**Figure 3:**
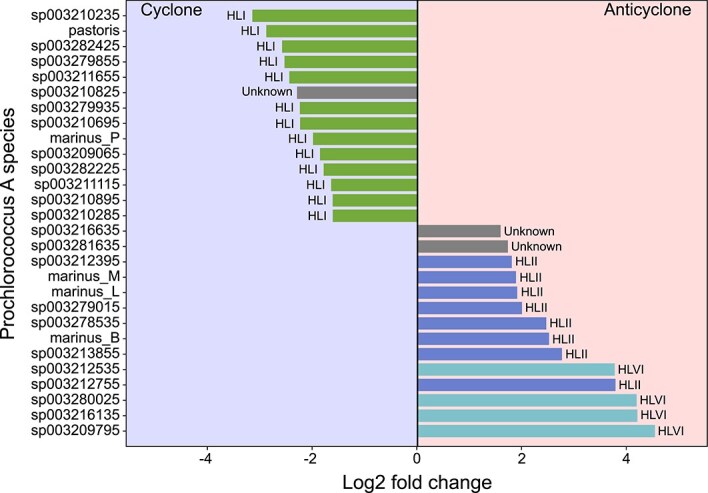
Differential representation of *Prochlorococcus* population variants between adjacent cyclonic versus anticyclonic eddies around the DCM. The MESO-SCOPE cruise DCM’s log2 fold change of transcript counts between the anticyclone and the cyclone is shown for species-level annotations. Fold change was calculated by dividing the mean of the expression sum value of the anticyclone by the cyclone for each species (n = 18 for each location). Only species with adjusted *P* values <0.05 and a fold change value higher than 3 for the anticyclone and smaller than ⅓ for the cyclone are shown. Statistical significance was calculated using the Kruskal-Wallis H-test. *P* values were corrected using the Benjamini-Hochberg procedure. Ecotype affiliation (adjacent to each bar head) according to genome to ITS mapping analysis is shown in Supplementary Fig. 4.

Summing transcriptional data by species and comparing it across DCM eddy environments, we identified a subset of *Prochlorococcus A* species exhibiting significant transcript enrichment in the cyclonic eddy (Kruskal-Wallis rank sum test, adjusted *P* values <0.05; ≥ three-fold difference in transcript read sums, n = 18 samples per eddy; Fig. 3). In the anticyclone, HLVI (4 species) and HLII (8 species), along with two unidentified HL species, were the most enriched. In contrast, 13 HLI-associated GTDB species and one unidentified HL species were overrepresented in the cyclone. Among these, the species *Prochlorococcus A pastoris* (a GTDB species representative of cultivated HLI strains MED4 and EQPAC1) was the second most enriched in the cyclone, contributing substantially to transcript counts at the cyclonic DCM (Supplementary Fig. 6). This comparative metatranscriptomics pattern aligns with our metagenomic analysis, which revealed HLI genome enrichment in the cyclone during the MESO-SCOPE expedition (Fig. 2), as well as the HLI enrichment above the DCM in cyclones observed in our 16S rRNA gene ASV analysis (Fig. 1).

We expanded this analysis to include HLI *Prochlorococcus A* species with a fold-change between the eddies greater than 2 (Supplementary Fig. 5). Among the 24 transcript-enriched species in the cyclone, 20 belonged to the HLI ecotype, three to an unknown HL ecotype, and only one to HLII. In contrast, none of the 35 species overrepresented in the anticyclone DCM were associated with the HLI ecotype (Supplementary Fig. 5). Instead, four species were affiliated with HLVI, 10 with an unknown HL ecotype, and 21 *Prochlorococcus A* species were annotated as HLII ecotype.

### Environmental conditions associated with *Prochlorococcus* HLI ecotype proliferation

During both cruises, the DCM was consistently shallower in cyclones than in anticyclones, with a difference of ~20 meters during MESO-SCOPE and over 40 meters during HOE-Legacy 4 [25]. Although cyclones had a shallower DCM than anticyclones, DCM temperatures were consistently lower in cyclones, averaging between 20.6°C ± 0.7 and 16.8°C ± 0.8 in cyclones, compared to 23.6°C ± 1.2 and 21.5°C ± 1.1 in anticyclones across both cruises [24, 25]. At the depth of the greatest HLI cyclone enrichment (Fig. 2A, 2B), we observed about a 3°C temperature difference (Fig. 2C). Our data is consistent with prior laboratory findings demonstrating distinct growth temperature optima for different *Prochlorococcus* ecotypes, as well as oceanographic transects showing that HLI ecotypes tend to thrive in cooler waters and temperate regions, whereas HLII prevails in warmer subtropical waters [39, 47, 48, 82–84]. Unlike these large-scale, persistent biogeographic and depth trends, the relatively short lifespan (weeks to months) and small horizontal spatial scale of mesoscale eddies introduce a different type of more transient environmental variability in the NPSG. The short-lived temperature gradients created by mesoscale eddies (Fig. 2C and Barone et al., 2022 [25]), like those reported here, appear to drive community reassembly and fine-scale niche partitioning within *Prochlorococcus* communities.

Temperature was not the only factor distinguishing the environments of adjacent eddies near the bottom of the photic zone. Higher photosynthetic active radiation (PAR) in the cyclone [24] potentially impacts *Prochlorococcus* community composition, selecting cells that thrive under higher light conditions and replacing part of the low-light adapted cells that generally thrive in the DCM. In combination with the elevated oxygen concentration measured [25], the environmental conditions above the cyclone DCM could select specifically for HLI (e.g. MED4) that is known to express an alternative oxidase gene (alternative quinol oxidases) to balance electron flow when adapting to increasing light levels [38, 85, 86]. A trait that restricts the growth of this strain in low oxygen (<2.5 μM) [38] but could be advantageous under the ~100 times higher oxygen levels detected in the cyclone above the DCM (240 μM) [25]. This cyclonic layer of high oxygen anomaly and increased productivity above the cyclone DCM coincides in depth with the peak abundance of *Prochlorococcus* HLI ecotypes, as observed in both 16S rRNA gene ASV survey and metagenomic analysis (Fig. 1 and Fig. 2). The differences in the environmental conditions between the eddies around the DCM depth raised the question of the specific conditions supporting the succession of *Prochlorococcus* HLI at and above the cyclone DCM.

### Cyclone-enriched *Prochlorococcus* HLI ecotype functional traits between adjacent cyclonic versus anticyclonic eddies

**Figure 4:**
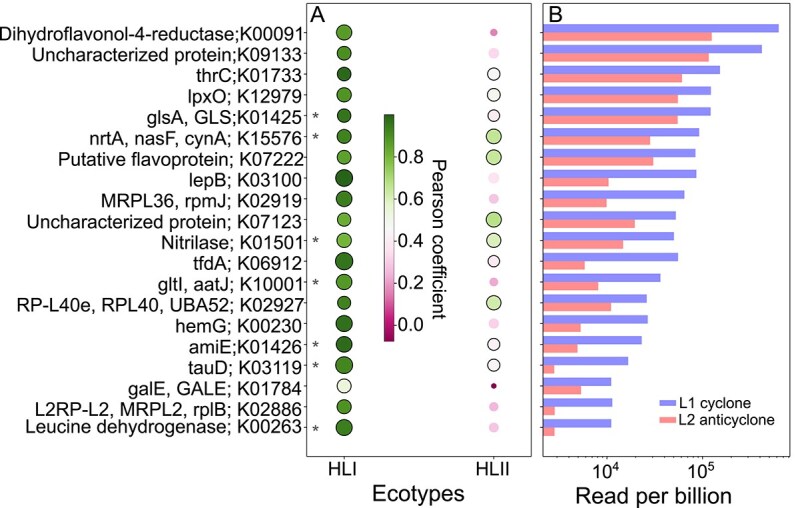
Correlation of cyclone-enriched genes with *Prochlorococcus* HLI ecotype distribution. KEGG Orthologs (KOs) with their respective Pearson correlation values (**A**, dot color and size) between gene reads per billion (RPB) count and specific ecotype by HLI/HLII ITS RPB counts from high-resolution depth survey around the DCM in cyclone and anticyclone during MESO-SCOPE cruise. All presented genes had HLI-correlated Pearson correlation values >0.50, a difference/total ratio ((cyclone-anticyclone)/total) of >0.50, and *P* values <0.05 (bold dot border). Gene Pearson correlation values with HLII ITS RPB are presented for comparison. KOs with functional annotations related to nitrogen metabolism are marked with asterisks (*). The sum of RPB in samples of the cyclone (blue bars) and anticyclone (red bars) for each gene (**B**). K00091; Dihydroflavonol-4-reductase[EC:1.1.1.219], K09133; uncharacterized protein, K01733; thrC; threonine synthase[EC:4.2.3.1], K12979; lpxO; beta-hydroxylase[EC:1.14.11.-], K01425; glsA; GLS; glutaminase[EC:3.5.1.2], K15576; nrtA; nasF; cynA; nitrate/nitrite transport system substrate-binding protein, K07222; putative flavoprotein involved in K+ transport, K03100; lepB; signal peptidase I[EC:3.4.21.89], K02919; MRPL36; rpmJ; large subunit ribosomal protein L36RP-L36, K07123; uncharacterized protein, K01501; nitrilase[EC:3.5.5.1], K01501; tfdA; alpha-ketoglutarate-dependent 2,4-dichlorophenoxyacetate dioxygenase[EC:1.14.11.-], K10001; gltI; aatJ; glutamate/aspartate transport system substrate-binding protein, K02927; RP-L40e; RPL40; UBA52; ubiquitin-large subunit ribosomal protein L40e, K00230; hemG; menaquinone-dependent protoporphyrinogen oxidase[EC:1.3.5.3], K01426; amiE; amidase[EC:3.5.1.4], K03119; tauD; taurine dioxygenase[EC:1.14.11.17], K01784; gale; GALE; UDP-glucose 4-epimerase[EC:5.1.3.2], K02886; L2RP-L2; MRPL2; rplB; large subunit ribosomal protein, K00263; leucine dehydrogenase[EC:1.4.1.9].

To investigate functional attributes of *Prochlorococcus* HLI enriched in the cyclonic eddy, we conducted metagenomic and metatranscriptome analyses on picoplankton samples collected from both cyclonic and anticyclonic DCMs during the MESO-SCOPE cruise. We focused the metagenomic analyses on *Prochlorococcus* HLI and HLII by mapping reads to known ecotypes and calculating Pearson correlations between gene abundances aggregated by Kyoto Encyclopedia of Genes and Genomes (KEGG) Orthology (KO) annotations and the average reads per billion (RPB) of *Prochlorococcus* HLI or HLII ecotypes in the MESO-SCOPE metagenomic dataset (Supplementary Table S5). Key genes enriched in the cyclone were assessed by comparing gene abundances between the cyclonic and anticyclonic eddies, highlighting 20 KOs most strongly correlated with HLI ecotypes, and sorting them based on relative abundances (Fig. 4). Among these genes were several (8 out of 20) involved in nitrogen acquisition and metabolism (*thrC*, *glsA*/*GLS*, *nrtA*/*nasF*/*cynA*, nitrilase (K01501), *gltI*/*aatJ*, *amiE*, *tauD*, and leucine dehydrogenase). These genes may indicate general metabolic differences between the cyclone-enriched HLI groups and the anticyclone *Prochlorococcus* population (Fig. 4). Several of these genes also had a significant positive correlation with *Prochlorococcus* HLII sequences, including *nrtA*/*nasF*/*cynA* (0.64, *P-*value <0.01) and a nitrilase gene (0.59, *P-*value <0.01), but were still enriched in the cyclone, along with the HLI ecotype (Fig. 4).

*Prochlorococcus* HLI and HLII ecotypes exhibit complex differences in nutrient-related genes, particularly for nitrogen metabolism. Although all *Prochlorococcus* can utilize ammonium and none can fix dinitrogen, they vary in their ability to assimilate other nitrogen sources, including oxidized inorganic nitrogen like nitrate and nitrite, and other reduced forms like urea, cyanate, and amino acids [40, 87–92]. Even within a single ecotype, different genomes exhibit variability in their potential to assimilate and reduce inorganic nitrogen [87]. Cross-feeding interactions involving nitrite have been proposed to occur at the base of the photic zone, just above the nutricline and DCM, where cells possessing the complete nitrate/nitrite assimilation pathway supply nitrite to cells of the LL ecotype that encode only the nitrite transporter and reductase [93]. Nitrate and nitrite assimilation genes (*napA*, *narB*, *nirA*, and *focA*) are absent in cultured HLI strains, rare in cultured HLII strains, yet present in HLI and HLII single-cell amplified and metagenome-assembled genomes, indicating substantial variability in this trait within and between ecotypes. All reported HLI and HLII genomes lacked the nitrite-specific transporter gene *focA*. Among HLI genomes, those carrying the nitrite reductase gene *nirA* also harbored the nitrate reductase (*narB*) and nitrate/nitrite transporter (*napA*) genes. In contrast, HLII genomes showed greater variability, with some missing only *nirA*, whereas others possessed *nirA* but lacked *napA* and *narB* [87].

Although sequenced genomes from both HLI and HLII ecotypes lacked the nitrite-specific transporter gene, *focA*, some contained the general nitrate/nitrite transporter *napA*. Our data further suggests the presence of the *nrtA/nasF/cynA* genes, which encode an ABC-type nitrate/nitrite transporter, as well as nitrate and nitrite reductases. These genetic features could support HLI ecotype growth under the conditions of the local nitrite maximum. Although HLI showed a higher correlation with *nrtA/nasF/cynA* genes in our metagenomic analysis, we could not attribute this advantage exclusively to HLI, as HLII may also possess and utilize these genes (Fig. 4).

We found significant correlations between specific functions and *Prochlorococcus* HLI metagenomic reads within the cyclonic DCM, supporting their specific adaptation to this unique environmental niche found in the cyclone. We further examined variations in gene expression between DCM populations of the two eddies, analyzing patterns of transcript abundance between cyclonic and anticyclonic eddies sampled during the MESO-SCOPE expedition. We focused specifically on transcripts associated with the *Prochlorococcus* HLI variants overrepresented in the cyclone's DCM (Supplementary Fig. 5). We identified 675 transcripts with statistically significant (Benjamini-Hochberg adjusted *P* values<0.05) differential expression between the eddies at the DCM (Supplementary Fig. 7). Among these, 423 transcripts matched known genes, providing functional annotations and metabolic pathway information on differentially expressed transcripts. The HLI-annotated gene transcripts displayed differential elevated representation in either the cyclone or the anticyclone, depending on the HLI strains from which they were derived (Supplementary Table S6 and Supplementary Table S7).

**Figure 5:**
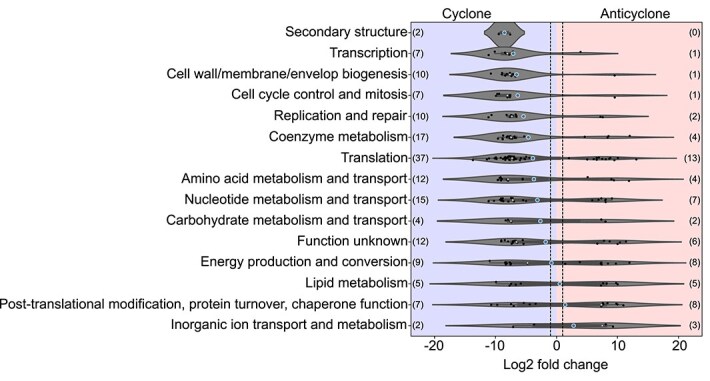
Differential representation of *Prochlorococcus* A HLI gene transcripts in adjacent cyclonic versus anticyclonic eddies. Violin plot illustrating transcription log_2_ fold change data for transcripts (n = 223 transcripts) of *Prochlorococcus* A HLI species overrepresented in the cyclone DCM, categorized by COG functional categories. Only transcripts with statistical significance (adjusted *P* values<0.05) and mean log_2_ fold change values greater than two or smaller than −2 are included. Positive log_2_ fold change values on the right correspond to enrichment in the anticyclonic eddy, whereas negative log_2_ fold change values on the left correspond to enrichment in the cyclonic eddy. The number of transcripts in each category for cyclone and anticyclone is given in parentheses (left or right side of the graph, respectively). A small white dot indicates the median. And the bigger blue dot indicates the mean. Black dots represent data points in a jitter plot. The dashed lines mark the −2 and 2 log_2_ fold change values.

To characterize specific *Prochlorococcus* HLI functions with varying expression levels between the eddies’ DCM, we examined the transcripts contributing to different gene annotations [94], excluding genes with multiple overexpressed transcripts in both eddies. This analysis yielded 223 distinct overexpressed transcripts from 190 unique eggNOG annotations, each showing significant overexpression in one eddy over the other. We then categorized these transcripts based on clusters of orthologous groups (COG) functional categories (Supplementary Table S8). Overexpressed HLI transcripts in the cyclone predominantly fell into categories related to cell replication and proliferation (transcription, cell wall/membrane/envelope biogenesis, cell cycle control and mitosis, DNA replication and repair, and protein translation), emphasizing the succession of HLI variants in this distinct environment (Fig. 5). In contrast, inorganic ion transport and metabolism, post-translational modification, protein turnover, and chaperone functions had a higher representation of overexpressed transcripts in the anticyclone. This indicates that the dominant functional categories overexpressed in the anticyclone are associated with nutrient acquisition and stress response (Fig. 5).

To further elucidate the functional differences expressed by the cyclone-enriched HLI between the eddies, we used KEGG orthology annotations to categorize the 675 overexpressed transcripts by pathways. Of these, 286 transcripts had identifiable KEGG orthology annotations, often linked to multiple KEGG pathways. We retained pathways having more than two overexpressed transcripts, eliminating less relevant or broadly defined categories. Additionally, we merged specific categories of relevant pathways (e.g. base excision repair and nucleotide excision repair) and excluded transcripts of ambiguous eggNOG gene annotations contributed by transcripts overexpressed in both eddies (Supplementary Table S9). The fold change data for transcripts in each pathway category were then visualized as a box plot (Supplementary Fig. 8). Among these, nine pathways had more transcripts overexpressed in the anticyclone, whereas four pathways showed an equal number of transcripts overexpressed in both eddies. Thirty-three pathway categories exhibited more transcripts being overexpressed in the cyclone. Common themes among pathways overexpressed in the anticyclone were related to basic physiological needs, environmental adaptation, and cellular conservation processes. This included energy production (oxidative phosphorylation and Photosynthesis), adaptation to environmental changes (two-component system, biofilm formation, and ABC transporters), and cellular conservation processes such as RNA degradation (Supplementary Fig. 8).

**Figure 6:**
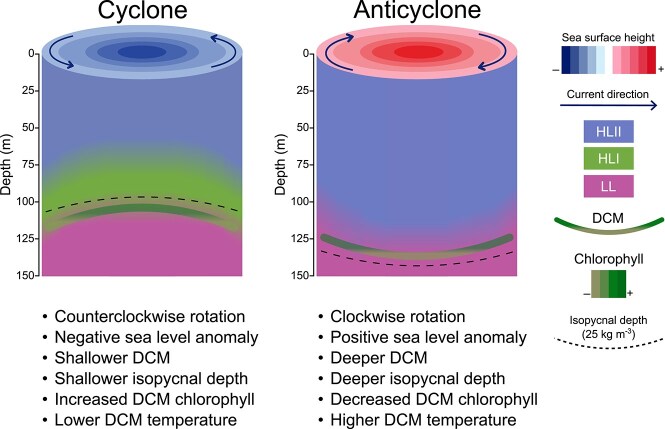
Illustration depicting the effect of eddies on *Prochlorococcus* HLI and HLII populations in the NPSG. The schematic contrasts cyclonic and anticyclonic eddies, highlighting differences in sea surface height, eddy current direction (in the Northern Hemisphere), and isopycnal displacement. In cyclonic eddies, the shallowing of the DCM and isopycnals, coupled with elevated nutrient supply and lower temperatures, promotes proliferation of the HLI *Prochlorococcus* ecotype near the DCM. In anticyclonic eddies, characterized by deeper DCM, deeper isopycnals, and warmer, nutrient-poor conditions, the HLII ecotype is dominant.

Metabolic pathways with high transcript abundances in the cyclone primarily pertained to cell replication, proliferation, and amino acid biosynthesis and metabolism. This included transcripts associated with DNA replication and repair, homologous recombination, RNA polymerase, ribosome, aminoacyl-tRNA biosynthesis, and quorum sensing (Supplementary Fig. 8). Others encompassed the production of cellular building blocks in pathways like carbon fixation, citrate cycle (TCA cycle), pentose phosphate pathway, pantothenate and CoA biosynthesis, pyrimidine and purine metabolism, as well as amino acid biosynthesis and metabolism in pathways like biosynthesis of amino acids, phenylalanine, tyrosine and tryptophan biosynthesis, lysine biosynthesis, cysteine and methionine metabolism, alanine, aspartate, and glutamate metabolism, glycine, serine, and threonine metabolism (Supplementary Fig. 8). The overexpression of amino acid transport, metabolism, and synthesis in the HLI cyclone population suggests the importance of nitrogen metabolism for the succession of *Prochlorococcus* HLI in the cyclone.

Further evidence for distinct nitrogen dynamics at the cyclone’s DCM emerged from community-wide transcript abundances, which reflected differential representation of prokaryotic genera between eddies. The most overrepresented genera belonged to Marine Group IIb archaea (MGIIb-O2 and MGIIb-O5, Supplementary Fig. 9), members of Thalassarchaeaceae known to metabolize amino acids and peptides for energy, potentially releasing nitrogenous byproducts such as ammonia or urea [95, 96]. Although direct ammonia measurements are unavailable, the concurrent overrepresentation of ammonia-oxidizing archaea (*Nitrosopelagicus*, *Nitrosotenuis*, and *Nitrosopumilus*, Supplementary Fig. 9) suggests active recycling and transformation of nitrogen at the DCM in the cyclone. These patterns indicate a dynamic nitrogen microenvironment that may support both heterotrophic remineralization and autotrophic nitrification, shaping nitrogen availability for the microbial community.

The observed shifts in *Prochlorococcus* ecotype distributions between adjacent eddies illustrate how transient physicochemical variability can restructure microbial communities in a stable and homogeneous oceanic regime. This is relevant to Hutchinson’s “paradox of the plankton,” which questioned how diverse phytoplankton communities can persist under seemingly uniform conditions [97]. One proposed resolution is that non-equilibrium dynamics continuously generate new niche opportunities [98]. In this context, mesoscale eddies function as dynamic disturbances that transiently reshape niche structures across short spatial and temporal scales. Previous studies suggest that much of the genomic and genetic diversity observed within *Prochlorococcus* communities is attributable to ecological selection across gradients of stable depth stratification, seasonal changes, or global latitudinal distributions [35, 47, 48, 50, 84, 99]. Transient, local-scale environmental variability associated with eddies, in contrast, creates ephemeral niches over relatively short time scales and in close horizontal spatial proximity. 

## Conclusions

In this study, we explored the differences in *Prochlorococcus* genetic variants between adjacent cyclonic and anticyclonic eddies. Our analyses revealed an enrichment of *Prochlorococcus* HLI variants within and above the DCM of cyclones (but not in anticyclones), which was associated with distinct environmental conditions. Beyond the differential community structure of *Prochlorococcus* populations observed between adjacent eddies having different polarities, we also examined the differences in *Prochlorococcus* functional gene traits across the eddies. The data suggest that the metabolic potential of *Prochlorococcus* communities, especially those associated with nitrogen metabolism, differs between cyclones and anticyclones. These differences were associated with the elevated representation of *Prochlorococcus* HLI variants in the cyclone. Metatranscriptomic analyses similarly revealed variations in *Prochlorococcus* gene expression patterns, with the cyclone having elevated transcript abundances in genes related to energy production, cell replication and proliferation, amino acid transport, metabolism, and biosynthesis.

Temperature emerged as one critical environmental factor, consistent with laboratory growth experiments using pure cultures of *Prochlorococcus* HLI and HLII strains. In addition to temperature, variations in light availability and nutrient concentrations [24, 25] likely contribute to the distinct ecological niches that differentiate the DCMs of adjacent eddies and drive the proliferation of specific *Prochlorococcus* variants (Fig. 6). Because all observations we report here occurred during spring and summer, further investigation will be required to determine if eddies that occur in winter and fall have similar effects on *Prochlorococcus* assemblages. Although NPSG surface waters are typically considered physically and biologically stable, our results reveal that mesoscale eddies introduce short-term environmental heterogeneity that can alter population structure, functional potential, and gene expression. These findings provide new insights into how transient oceanographic features generate niche diversity and promote the coexistence of closely related microbial populations in oligotrophic ocean ecosystems.

## Supplementary Material

Supplemental_Material_text_and_figures_wraf106

Supplementary_tables_Syen_et_al_20250417_wraf106

## Data Availability

16S rRNA gene amplicon sequences from the MESO-SCOPE (KM1709) and HOE-Legacy 4 (KOK1607) cruises are available at https://www.ncbi.nlm.nih.gov/bioproject/PRJNA596510 and https://www.ncbi.nlm.nih.gov/bioproject/707586, respectively. Cloned 16S rRNA-ITS-23S rRNAs from the MESO-SCOPE (KM1709) cruise are available at https://www.ncbi.nlm.nih.gov/bioproject/PRJNA596510. Metagenome depth profiles in the eddies from the MESO-SCOPE (KM1709) cruise are available at https://www.ncbi.nlm.nih.gov/bioproject/PRJNA596510. Metatranscriptomes from 15 m and the DCM in the eddies from the MESO-SCOPE (KM1709) cruise are available at https://www.ncbi.nlm.nih.gov/bioproject/PRJNA596510. MESO-SCOPE (KM1709) environmental metadata are available at http://scope.soest.hawaii.edu/data/mesoscope/mesoscope.html. HOE-Legacy 4 (KOK1607) environmental metadata are available at http://scope.soest.hawaii.edu/data/hoelegacy/documents. Count values tables for metagenomes, and metatranscriptomes, as well as internal standard and volume conversion factor tables for use in the normalization of metatranscriptome raw counts, are available in the open public database Zenodo https://doi.org/10.5281/zenodo.15178202.

## References

[1] Karl DM, Winn CA. A sea of change: monitoring the oceans’ carbon cycle. *Environ Sci Technol* 1991;25:1977–81. 10.1021/es00024a600

[2] Karl DM, Lukas R. The Hawaii Ocean time-series (HOT) program: background, rationale and field implementation. *Deep Sea Res Part II Top Stud Oceanogr* 1996;43:129–56. 10.1016/0967-0645(96)00005-7

[3] Karl DM, Church MJ. Station ALOHA: a gathering place for discovery, education, and scientific collaboration. *Limnol Oceanogr Bull* 2019;28:10–2. 10.1002/lob.10285

[4] Karl DM, Letelier RM, Bidigare RR. et al. Seasonal-to-decadal scale variability in primary production and particulate matter export at station ALOHA. *Prog Oceanogr* 2021;195:102563. 10.1016/j.pocean.2021.102563

[5] Viviani DA, Church MJ. Decoupling between bacterial production and primary production over multiple time scales in the North Pacific subtropical gyre. *Deep Sea Res Part I Oceanogr Res Pap* 2017;121:132–42. 10.1016/j.dsr.2017.01.006

[6] Karl DM, Church MJ. Ecosystem structure and dynamics in the North Pacific subtropical gyre: new views of an old ocean. *Ecosystems.* 2017;20:433–57. 10.1007/s10021-017-0117-0

[7] Karl DM, Letelier RM, Tupas L. et al. The role of nitrogen fixation in biogeochemical cycling in the subtropical North Pacific Ocean. *Nature.* 1997;388:533–8. 10.1038/41474

[8] Knorr LACM, Sabine CL, Sutton AJ. et al. Quantifying net community production and calcification at station ALOHA near Hawai’i: insights and limitations from a dual tracer carbon budget approach. *Glob Biogeochem Cycles* 2023;37:e2022GB007672. 10.1029/2022GB007672

[9] Coale KH, Bruland KW. Oceanic stratified euphotic zone as elucidated by ^234^Th:^238^U disequilibria. *Limnol Oceanogr* 1987;32:189–200. 10.4319/lo.1987.32.1.0189

[10] Eppley RW, Renger EH, Venrick EL. et al. A study of plankton dynamics and nutrient cycling in the central gyre of the North Pacific Ocean. *Limnol Oceanogr* 1973;18:534–51. 10.4319/lo.1973.18.4.0534

[11] Small LF, Knauer GA, Tuel MD. The role of sinking fecal pellets in stratified euphotic zones. *Deep Sea Res Part I Oceanogr Res Pap.* 1987;34:1705–11. 10.1016/0198-0149(87)90019-7

[12] Dugdale RC . Nutrient limitation in the sea: dynamics, identification, and significance. *Limnol Oceanogr* 1967;12:685–95. 10.4319/lo.1967.12.4.0685

[13] Böttjer D, Dore JE, Karl DM. et al. Temporal variability of nitrogen fixation and particulate nitrogen export at station ALOHA. *Limnol Oceanogr* 2017;62:200–16. 10.1002/lno.10386

[14] Johnson KS, Riser SC, Karl DM. Nitrate supply from deep to near-surface waters of the North Pacific subtropical gyre. *Nature.* 2010;465:1062–5. 10.1038/nature09170 20577212

[15] Benitez-Nelson CR, Bidigare RR, Dickey TD. et al. Mesoscale eddies drive increased silica export in the subtropical Pacific Ocean. *Science.* 2007;316:1017–21. 10.1126/science.1136221 17510362

[16] Brown SL, Landry MR, Selph KE. et al. Diatoms in the desert: plankton community response to a mesoscale eddy in the subtropical North Pacific. *Deep Sea Res Part II Top Stud Oceanogr* 2008;55:1321–33. 10.1016/j.dsr2.2008.02.012

[17] Bibby TS, Gorbunov MY, Wyman KW. et al. Photosynthetic community responses to upwelling in mesoscale eddies in the subtropical North Atlantic and Pacific oceans. *Deep Sea Res Part II Top Stud Oceanogr.* 2008;55:1310–20. 10.1016/j.dsr2.2008.01.014

[18] Bibby TS, Moore CM. Silicate:nitrate ratios of upwelled waters control the phytoplankton community sustained by mesoscale eddies in sub-tropical North Atlantic and Pacific. *Biogeosciences.* 2011;8:657–66. 10.5194/bg-8-657-2011

[19] Chen YL, Chen HY, Jan S. et al. Biologically active warm-core anticyclonic eddies in the marginal seas of the western Pacific Ocean. *Deep Sea Res Part I Oceanogr Res Pap.* 2015;106:68–84. 10.1016/j.dsr.2015.10.006

[20] Barone B, Coenen AR, Beckett SJ. et al. The ecological and biogeochemical state of the North Pacific subtropical gyre is linked to sea surface height. *J Mar Res* 2019;77:215–45. 10.1357/002224019828474241

[21] Xiu P, Chai F. Eddies affect subsurface phytoplankton and oxygen distributions in the North Pacific subtropical gyre. *Geophys Res Lett* 2020;47:e2020GL087037. 10.1029/2020GL087037

[22] Dugenne M, Henderikx Freitas F, Wilson ST. et al. Life and death of *Crocosphaera* sp. in the Pacific Ocean: fine scale predator–prey dynamics. *Limnol Oceanogr* 2020;65:2603–17. 10.1002/lno.11473

[23] Henderikx Freitas F, Dugenne M, Ribalet F. et al. Diel variability of bulk optical properties associated with the growth and division of small phytoplankton in the North Pacific subtropical gyre. *Appl Opt* 2020;59:6702–16. 10.1364/AO.394123 32749375

[24] Hawco NJ, Barone B, Church MJ. et al. Iron depletion in the deep chlorophyll maximum: mesoscale eddies as natural iron fertilization experiments. *Glob Biogeochem Cycles* 2021;35:e2021GB007112. 10.1029/2021GB007112

[25] Barone B, Church MJ, Dugenne M. et al. Biogeochemical dynamics in adjacent mesoscale eddies of opposite polarity. *Glob Biogeochem Cycles* 2022;36:e2021GB007115. 10.1029/2021GB007115

[26] Dugenne M, Gradoville MR, Church MJ. et al. Nitrogen fixation in mesoscale eddies of the North Pacific subtropical gyre: patterns and mechanisms. *Glob Biogeochem Cycles* 2023;37:e2022GB007386. 10.1029/2022GB007386

[27] McGillicuddy DJ, Robinson AR, Siegel DA. et al. Influence of mesoscale eddies on new production in the Sargasso Sea. *Nature.* 1998;394:263–6. 10.1038/28367

[28] McGillicuddy DJ, Anderson LA, Bates NR. et al. Eddy/wind interactions stimulate extraordinary mid-ocean plankton blooms. *Science.* 2007;316:1021–6. 10.1126/science.1136256 17510363

[29] McGillicuddy DJ, Robinson AR. Eddy-induced nutrient supply and new production in the Sargasso Sea. *Deep Sea Res Part I Oceanogr Res Pap* 1997;44:1427–50. 10.1016/S0967-0637(97)00024-1

[30] Cornec M, Laxenaire R, Speich S. et al. Impact of mesoscale eddies on deep chlorophyll maxima. *Geophys Res Lett* 2021;48:e2021GL093470. 10.1029/2021GL093470 PMC8365668 34433995

[31] McGillicuddy DJ . Mechanisms of physical–biological–biogeochemical interaction at the oceanic mesoscale. *Annu Rev Mar Sci* 2016;8:125–59. 10.1146/annurev-marine-010814-015606 26359818

[32] Beatty JL, Stewart BP, Mesrop LY. et al. Eddy dipole differentially influences particle-associated and water column protistan community composition. *Limnol Oceanogr* 2025;70:817–32. 10.1002/lno.12785

[33] Huang J, Xu F. Observational evidence of subsurface chlorophyll response to mesoscale eddies in the North Pacific. *Geophys Res Lett* 2018;45:8462–70. 10.1029/2018GL078408

[34] Nelson CE, Carlson CA, Ewart CS. et al. Community differentiation and population enrichment of Sargasso Sea bacterioplankton in the euphotic zone of a mesoscale mode-water eddy. *Environ Microbiol* 2014;16:871–87. 10.1111/1462-2920.12241 24589288

[35] Rocap G, Larimer FW, Lamerdin J. et al. Genome divergence in two *Prochlorococcus* ecotypes reflects oceanic niche differentiation. *Nature.* 2003;424:1042–7. 10.1038/nature01947 12917642

[36] Moore LR, Rocap G, Chisholm SW. Physiology and molecular phylogeny of coexisting *Prochlorococcus* ecotypes. *Nature.* 1998;393:464–7. 10.1038/30965 9624000

[37] Ulloa O, Henríquez-Castillo C, Ramírez-Flandes S. et al. The cyanobacterium *Prochlorococcus* has divergent light-harvesting antennae and may have evolved in a low-oxygen ocean. *Proc Natl Acad Sci USA* 2021;118:e2025638118. 10.1073/pnas.2025638118 33707213 PMC7980375

[38] Savoie M, Mattison A, Genge L. et al. *Prochlorococcus marinus* responses to light and oxygen. *PLoS One* 2024;19:e0307549. 10.1371/journal.pone.0307549 39038009 PMC11262661

[39] Johnson ZI, Zinser ER, Coe A. et al. Niche partitioning among *Prochlorococcus* ecotypes along ocean-scale environmental gradients. *Science.* 2006;311:1737–40. 10.1126/science.1118052 16556835

[40] Moore LR, Post AF, Rocap G. et al. Utilization of different nitrogen sources by the marine cyanobacteria *Prochlorococcus* and *Synechococcus*. *Limnol Oceanogr* 2002;47:989–96. 10.4319/lo.2002.47.4.0989

[41] Moore LR, Ostrowski M, Scanlan DJ. et al. Ecotypic variation in phosphorus-acquisition mechanisms within marine picocyanobacteria. *Aquat Microb Ecol* 2005;39:257–69. 10.3354/ame039257

[42] Veaudor T, Cassier-Chauvat C, Chauvat F. Genomics of urea transport and catabolism in cyanobacteria: biotechnological implications. *Front Microbiol* 2019;10:02052. 10.3389/fmicb.2019.02052 PMC6737895 31551986

[43] Zubkov MV, Tarran GA. Amino acid uptake of *Prochlorococcus* spp. in surface waters across the South Atlantic subtropical front. *Aquat Microb Ecol* 2005;40:241–9. 10.3354/ame040241

[44] Zubkov MV, Tarran GA, Fuchs BM. Depth-related amino acid uptake by *Prochlorococcus* cyanobacteria in the southern Atlantic tropical gyre. *FEMS Microbiol Ecol* 2004;50:153–61. 10.1016/j.femsec.2004.06.009 19712356

[45] Morel A, Ahn YH, Partensky F. et al. *Prochlorococcus* and *Synechococcus*: a comparative study of their optical properties in relation to their size and pigmentation. *J Mar Res* 1993;51:617–49. 10.1357/0022240933223963

[46] Raven JA . Why are there no picoplanktonic O_2_ evolvers with volumes less than 10^−19^ m^3^? *J Plankton Res* 1994;16:565–80. 10.1093/plankt/16.5.565

[47] Malmstrom RR, Coe A, Kettler GC. et al. Temporal dynamics of *Prochlorococcus* ecotypes in the Atlantic and Pacific oceans. *ISME J.* 2010;4:1252–64. 10.1038/ismej.2010.60 20463762

[48] Biller SJ, Berube PM, Lindell D. et al. *Prochlorococcus*: the structure and function of collective diversity. *Nat Rev Microbiol* 2015;13:13–27. 10.1038/nrmicro3378 25435307

[49] Delmont TO, Eren AM. Linking pangenomes and metagenomes: the *Prochlorococcus* metapangenome. *PeerJ.* 2018;6:e4320. 10.7717/peerj.4320 29423345 PMC5804319

[50] Thompson AW, van den Engh GJ, Ahlgren NA. et al. Dynamics of *Prochlorococcus* diversity and photoacclimation during short-term shifts in water column stratification at station ALOHA. *Front Mar Sci* 2018;5:00488. 10.3389/fmars.2018.00488

[51] van den Engh GJ, Doggett JK, Thompson AW. et al. Dynamics of *Prochlorococcus* and *Synechococcus* at station ALOHA revealed through flow cytometry and high-resolution vertical sampling. *Front Mar Sci* 2017;4:359. 10.3389/fmars.2017.00359

[52] Luo E, Eppley JM, Romano AE. et al. Double-stranded DNA virioplankton dynamics and reproductive strategies in the oligotrophic open ocean water column. *ISME J* 2020;14:1304–15. 10.1038/s41396-020-0604-8 32060418 PMC7174320

[53] Parada AE, Needham DM, Fuhrman JA. Every base matters: assessing small subunit rRNA primers for marine microbiomes with mock communities, time series and global field samples. *Environ Microbiol* 2016;18:1403–14. 10.1111/1462-2920.13023 26271760

[54] Caporaso JG, Lauber CL, Walters WA. et al. Global patterns of 16S rRNA diversity at a depth of millions of sequences per sample. *Proc Natl Acad Sci USA* 2011;108 Suppl 1:4516–22. 10.1073/pnas.1000080107 20534432 PMC3063599

[55] Apprill A, McNally S, Parsons R. et al. Minor revision to V4 region SSU rRNA 806R gene primer greatly increases detection of SAR11 bacterioplankton. *Aquat Microb Ecol* 2015;75:129–37. 10.3354/ame01753

[56] Bolyen E, Rideout JR, Dillon MR. et al. Reproducible, interactive, scalable and extensible microbiome data science using QIIME 2. *Nat Biotechnol* 2019;37:852–7. 10.1038/s41587-019-0209-9 31341288 PMC7015180

[57] Callahan BJ, McMurdie PJ, Rosen MJ. et al. DADA2: high-resolution sample inference from Illumina amplicon data. *Nat Methods* 2016;13:581–3. 10.1038/nmeth.3869 27214047 PMC4927377

[58] Quast C, Pruesse E, Yilmaz P. et al. The SILVA ribosomal RNA gene database project: improved data processing and web-based tools. *Nucleic Acids Res* 2013;41:D590–6. 10.1093/nar/gks1219 23193283 PMC3531112

[59] McNichol J . ProPortal-ASV-Annotation. 2023 [cited 2025 May 02]. https://github.com/jcmcnch/ProPortal-ASV-Annotation.

[60] BBMap. SourceForge; 2024 [cited 2025 May 02]. https://sourceforge.net/projects/bbmap

[61] Li H . BFC: correcting Illumina sequencing errors. *Bioinformatics.* 2015;31:2885–7. 10.1093/bioinformatics/btv290 25953801 PMC4635656

[62] Bolger AM, Lohse M, Usadel B. Trimmomatic: a flexible trimmer for Illumina sequence data. *Bioinformatics.* 2014;30:2114–20. 10.1093/bioinformatics/btu170 24695404 PMC4103590

[63] Li D, Liu CM, Luo R. et al. MEGAHIT: an ultra-fast single-node solution for large and complex metagenomics assembly via succinct de Bruijn graph. *Bioinformatics.* 2015;31:1674–6. 10.1093/bioinformatics/btv033 25609793

[64] Li H . Aligning sequence reads, clone sequences and assembly contigs with BWA-MEM. arXiv [Preprint]. 2013;arXiv:1303.3997. 10.48550/arXiv.1303.3997

[65] Parks DH, Chuvochina M, Chaumeil PA. et al. A complete domain-to-species taxonomy for Bacteria and Archaea. *Nat Biotechnol* 2020;38:1079–86. 10.1038/s41587-020-0501-8 32341564

[66] Kanehisa M, Goto S. KEGG: Kyoto encyclopedia of genes and genomes. *Nucleic Acids Res* 2000;28:27–30. 10.1093/nar/28.1.27 10592173 PMC102409

[67] Bushmanova E, Antipov D, Lapidus A. et al. rnaSPAdes: a de novo transcriptome assembler and its application to RNA-Seq data. *GigaScience.* 2019;8:giz100. 10.1093/gigascience/giz100 31494669 PMC6736328

[68] Hyatt D, Chen GL, LoCascio PF. et al. Prodigal: prokaryotic gene recognition and translation initiation site identification. *BMC Bioinformatics* 2010;11:119. 10.1186/1471-2105-11-119 20211023 PMC2848648

[69] Fu L, Niu B, Zhu Z. et al. CD-HIT: accelerated for clustering the next-generation sequencing data. *Bioinformatics.* 2012;28:3150–2. 10.1093/bioinformatics/bts565 23060610 PMC3516142

[70] Parks DH, Chuvochina M, Waite DW. et al. A standardized bacterial taxonomy based on genome phylogeny substantially revises the tree of life. *Nat Biotechnol* 2018;36:996–1004. 10.1038/nbt.4229 30148503

[71] Allen LZ, Allen EE, Badger JH. et al. Influence of nutrients and currents on the genomic composition of microbes across an upwelling mosaic. *ISME J.* 2012;6:1403–14. 10.1038/ismej.2011.201 22278668 PMC3379637

[72] Kiełbasa SM, Wan R, Sato K. et al. Adaptive seeds tame genomic sequence comparison. *Genome Res* 2011;21:487–93. 10.1101/gr.113985.110 21209072 PMC3044862

[73] Cantalapiedra CP, Hernández-Plaza A, Letunic I. et al. eggNOG-mapper v2: functional annotation, orthology assignments, and domain prediction at the metagenomic scale. *Mol Biol Evol* 2021;38:5825–9. 10.1093/molbev/msab293 34597405 PMC8662613

[74] Virtanen P, Gommers R, Oliphant TE. et al. SciPy 1.0: fundamental algorithms for scientific computing in Python. *Nat Methods* 2020;17:261–72. 10.1038/s41592-019-0686-2 32015543 PMC7056644

[75] Seabold S, Perktold J . statsmodels: Econometric and statistical modeling with Python. In: van der Walt S, Millman J (eds). 9th Python in Science Conference, 28 Jun–3 Jul 2010. Austin, TX: SciPy; 2010. 10.25080/Majora-92bf1922-011

[76] Muzellec B, Teleńczuk M, Cabeli V, Andreux M . PyDESeq2: A Python package for bulk RNA-seq differential expression analysis. Bioinformatics. 2023;39:btad547. 10.1093/bioinformatics/btad547 PMC10502239 37669147

[77] Kelly L, Huang KH, Ding H. et al. ProPortal: a resource for integrated systems biology of *Prochlorococcus* and its phage. *Nucleic Acids Res* 2012;40:D632–40. 10.1093/nar/gkr1022 22102570 PMC3245167

[78] Rocap G, Distel DL, Waterbury JB. et al. Resolution of *Prochlorococcus* and *Synechococcus* ecotypes by using 16S-23S ribosomal DNA internal transcribed spacer sequences. *Appl Environ Microbiol* 2002;68:1180–91. 10.1128/AEM.68.3.1180-1191.2002 11872466 PMC123739

[79] Biller SJ, Berube PM, Lindell D. et al. Genomes of diverse isolates of the marine cyanobacterium *Prochlorococcus*. *Sci Data* 2014;1:140034. 10.1038/sdata.2014.34 25977791 PMC4421930

[80] Poff KE . Dynamics of Planktonic and Sinking Particle-Associated Microbes in the North Pacific Subtropical Gyre. Honolulu (HI): University of Hawai’i at Manoa; 2021. http://hdl.handle.net/10125/81646

[81] Parks DH, Chuvochina M, Rinke C. et al. GTDB: an ongoing census of bacterial and archaeal diversity through a phylogenetically consistent, rank normalized and complete genome-based taxonomy. *Nucleic Acids Res* 2022;50:D785–94. 10.1093/nar/gkab776 34520557 PMC8728215

[82] Zinser ER, Coe A, Johnson ZI. et al. *Prochlorococcus* ecotype abundances in the North Atlantic Ocean as revealed by an improved quantitative PCR method. *Appl Environ Microbiol* 2006;72:723–32. 10.1128/AEM.72.1.723-732.2006 16391112 PMC1352191

[83] Chandler JW, Lin Y, Gainer PJ. et al. Variable but persistent coexistence of *Prochlorococcus* ecotypes along temperature gradients in the ocean’s surface mixed layer. *Environ Microbiol Rep* 2016;8:272–84. 10.1111/1758-2229.12378 26743532

[84] Larkin AA, Blinebry SK, Howes C. et al. Niche partitioning and biogeography of high light adapted *Prochlorococcus* across taxonomic ranks in the North Pacific. *ISME J.* 2016;10:1555–67. 10.1038/ismej.2015.244 26800235 PMC4918451

[85] McDonald AE, Amirsadeghi S, Vanlerberghe GC. Prokaryotic orthologues of mitochondrial alternative oxidase and plastid terminal oxidase. *Plant Mol Biol* 2003;53:865–76. 10.1023/B:PLAN.0000023669.79465.d2 15082931

[86] Berg GM, Shrager J, van Dijken G. et al. Responses of *psbA*, *hli* and *ptox* genes to changes in irradiance in marine *Synechococcus* and *Prochlorococcus*. *Aquat Microb Ecol* 2011;65:1–14. 10.3354/ame01528

[87] Berube PM, Rasmussen A, Braakman R. et al. Emergence of trait variability through the lens of nitrogen assimilation in *Prochlorococcus*. *eLife.* 2019;8:e41043. 10.7554/eLife.41043 30706847 PMC6370341

[88] Berube PM, Biller SJ, Kent AG. et al. Physiology and evolution of nitrate acquisition in *Prochlorococcus*. *ISME J.* 2015;9:1195–207. 10.1038/ismej.2014.211 25350156 PMC4409163

[89] Zubkov MV, Fuchs BM, Tarran GA. et al. High rate of uptake of organic nitrogen compounds by *Prochlorococcus* cyanobacteria as a key to their dominance in oligotrophic oceanic waters. *Appl Environ Microbiol* 2003;69:1299–304. 10.1128/AEM.69.2.1299-1304.2003 12571062 PMC143617

[90] García-Fernández JM, de Marsac NT, Diez J. Streamlined regulation and gene loss as adaptive mechanisms in *Prochlorococcus* for optimized nitrogen utilization in oligotrophic environments. *Microbiol Mol Biol Rev* 2004;68:630–8. 10.1128/MMBR.68.4.630-638.2004 15590777 PMC539009

[91] Martiny AC, Kathuria S, Berube PM. Widespread metabolic potential for nitrite and nitrate assimilation among *Prochlorococcus* ecotypes. *Proc Natl Acad Sci USA* 2009;106:10787–92. 10.1073/pnas.0902532106 19549842 PMC2705535

[92] Kamennaya NA, Post AF. Characterization of cyanate metabolism in marine *Synechococcus* and *Prochlorococcus* spp. *Appl Environ Microbiol* 2011;77:291–301. 10.1128/AEM.01272-10 21057026 PMC3019706

[93] Berube PM, O’Keefe TJ, Rasmussen A. et al. Production and cross-feeding of nitrite within *Prochlorococcus* populations. *mBio.* 2023;14:e01236–23. 10.1128/mBio.01236-23 37404012 PMC10470740

[94] Huerta-Cepas J, Szklarczyk D, Heller D. et al. eggNOG 5.0: a hierarchical, functionally and phylogenetically annotated orthology resource based on 5090 organisms and 2502 viruses. *Nucleic Acids Res* 2019;47:D309–14. 10.1093/nar/gky1085 30418610 PMC6324079

[95] Rinke C, Rubino F, Messer LF. et al. A phylogenomic and ecological analysis of the globally abundant marine group II archaea (Candidatus Poseidoniales Ord. Nov.). *ISME J* 2019;13:663–75. 10.1038/s41396-018-0282-y 30323263 PMC6461757

[96] Tully BJ . Metabolic diversity within the globally abundant marine group II Euryarchaea offers insight into ecological patterns. *Nat Commun* 2019;10:271. 10.1038/s41467-018-07840-4 30655514 PMC6336850

[97] Hutchinson GE . The paradox of the plankton. *Am Nat* 1961;95:137–45. https://www.jstor.org/stable/2458386.

[98] MacArthur R, Levins R. The limiting similarity, convergence, and divergence of coexisting species. *Am Nat* 1967;101:377–85. 10.1086/282505

[99] Kashtan N, Roggensack SE, Rodrigue S. et al. Single-cell genomics reveals hundreds of coexisting subpopulations in wild *Prochlorococcus*. *Science.* 2014;344:416–20. 10.1126/science.1248575 24763590

