# Supplementary material for: Mesoscale eddies shape Prochlorococcus community structure and dynamics in the oligotrophic open ocean
Source: ISME J. 2025 May 24;19(1):wraf106. doi: 10.1093/ismejo/wraf106 (PMC12236433; doi:10.1093/ismejo/wraf106)
Supplement: Supplemental_Material_text_and_figures_wraf106 [file supplemental_material_text_and_figures_wraf106.pdf]

Supplementary information for

Mesoscale eddies shape *Prochlorococcus* community structure and dynamics in the oligotrophic open ocean

Uri Sheyn[1,2,*], Kirsten E Poff[1,*], John M Eppley[1], Andy O Leu[1,3], Jessica A Bryant[1,4], Fuyan Li[1], Anna E Romano[1,5], Andy Burger[1], Benedetto Barone[1], Edward F DeLong[1,ǂ].

*These authors contributed equally to this work.

[1]Daniel K. Inouye Center for Microbial Oceanography: Research and Education, University of Hawai'i, Honolulu, Hawai'i, USA.

[2]Department of Biological Sciences, Virginia Tech, Blacksburg, Virginia, USA.

[3]Centre for Microbiome Research, School of Biomedical Sciences, Queensland University of Technology (QUT), Translational Research Institute, Woolloongabba, Australia.

[4]Seres Therapeutics, Cambridge, Massachusetts, USA.

[5] California Institute of Technology, Pasadena, California, USA.

[ǂ] Corresponding author: Edward F. DeLong, Department of Oceanography, University of Hawai`i at Manoa, 1000 Pope Road, Honolulu, HI 96822, USA

Competing interest statement:

The authors declare no competing interests. This work was supported by the Simons Foundation through grants #329108, #721223, and #721264, and the Simons Foundation International grant #00010668.

Supplementary information

Files list:

A PDF file containing all supplementary text and figures.

Excel file containing all supplementary tables:

Supplementary Table 1; ST1 16S ASVs
Supplementary Table 2; ST3 ProPortal 16S – ecotype
Supplementary Table 3; ST4 16S stat tests
Supplementary Table 4; ST5 ITS to GTDB
Supplementary Table 5; ST6 gene to ecotype Pearson
Supplementary Table 6; ST7 MetaT COG cyclone
Supplementary Table 7; ST8 MetaT COG anticyclone
Supplementary Table 8; ST9 metaT COG eddies sum
Supplementary Table 9; ST10 MetaT KEGG pathway sum

Supplementary Tables list (Excel file)

Supplementary Table 1; ST1 16S ASVs

ASV-to-ecotype

Description: *Prochlorococcus* ecotype annotations for 16S rRNA amplicon sequence variants (ASVs) identified in this study. ASVs were annotated using IMG ProPortal and validated via phylogenetic analysis of Sanger-sequenced 16S-ITS clones. Associated Figure(s): Main Figure 1, Supplementary Figures 1–2.

Supplementary Table 2; ST2 16S stat tests

Statistical enrichment of ASV16 (HLI) across eddy types

Description: Statistical summary of Kruskal-Wallis and Wilcoxon rank sum tests comparing ASV16 (HLI) relative abundance across eddy types (cyclone, anticyclone, front) and cruises (MESO-SCOPE and HOE-Legacy 4). Associated Figure(s): Main Figure 1.

Supplementary Table 3; ST3 ProPortal 16S - ecotype

ProPortal ecotype annotation of 16S ASVs

Description: Ecotype assignments for 16S rRNA ASVs (ASV1–ASV137) including multiple HLII, HLI, and LL *Prochlorococcus* types as well as Synechococcus, based on IMG ProPortal and validated with 16S-ITS Sanger sequencing. Associated Figure(s): Main Figure 1, Supplementary Figure 1-2.

Supplementary Table 4; ST4 ITS to GTDB

ITS-based mapping of MAGs to ecotype and GTDB taxonomy

Description: Mapping of *Prochlorococcus* metagenome-assembled genomes (MAGs) to ecotype

designations and GTDB taxonomy, based on ITS region similarity and in silico PCR. Associated Figure(s): Supplementary Figure 4.

Supplementary Table 5; ST5 gene to ecotype Pearson cor

KO gene correlation with ecotype abundance

Description: Pearson correlation analysis between KEGG Orthology (KO) functional gene categories and the average metagenomic abundance of *Prochlorococcus* HLI and HLII ecotypes. Includes only genes with correlation coefficient and difference/total ratio > 0.5 and P values < 0.01. Associated Figure(s): Main Figure 4.

Supplementary Table 6; ST6 MetaT COG cyclone

Cyclone-enriched COG categories

Description: COG (eggNOG) functional categories for *Prochlorococcus* transcripts significantly overexpressed in the cyclone DCM (adjusted P < 0.05). Focused on HLI variants overrepresented in the cyclone. Associated Figure(s): Main Figure 5.

Supplementary Table 7; ST7 MetaT COG anticyclone

Anticyclone enriched COG categories.

Description: COG (eggNOG) functional categories for *Prochlorococcus* transcripts significantly overexpressed in the anticyclone DCM (adjusted P < 0.05). Focused on HLI variants overrepresented in the cyclone. Associated Figure(s): Main Figure 5.

Supplementary Table 8 ; ST8 metaT COG eddies sum

COG categories and overexpressed transcripts count in each eddy.

Description: COG functional categories of differentially expressed transcripts significantly overexpressed between DCM samples (adjusted P < 0.05), annotated using eggNOG preferred name. Median Log2 Fold Change between the eddies, and a number of transcripts overexpressed in each eddy. Overexpression data after removal of eggNOG annotation with transcripts overexpressed in both eddies. Associated Figure(s): Supplementary Figure 7, Main Figure 5.

Supplementary Table 9 ; ST9 MetaT KEGG pathway sum

KEGG pathways and overexpressed transcripts count in each eddy.

Description: KEGG pathways for differentially expressed transcripts (adjusted P < 0.05) between cyclone and anticyclone DCM samples, with median log2 fold change values, number of overexpressed transcripts in each eddy and eggNOG preferred names. Associated Figure(s): Supplementary Figure 8.

Additional Data

16S rRNA gene amplicon sequences from the MESO-SCOPE (KM1709) and HOE-Legacy 4 (KOK1607) cruises are available at https://www.ncbi.nlm.nih.gov/bioproject/PRJNA596510 and https://www.ncbi.nlm.nih.gov/bioproject/707586, respectively.

Cloned 16S rRNA-ITS-23S rRNAs from the MESO-SCOPE (KM1709) cruise are available at https://www.ncbi.nlm.nih.gov/bioproject/PRJNA596510.

Metagenome depth profiles in the eddies from the MESO-SCOPE (KM1709) cruise are available at https://www.ncbi.nlm.nih.gov/bioproject/PRJNA596510.

Metatranscriptomes from 15 m and the DCM in the eddies from the MESO-SCOPE (KM1709) cruise are available at https://www.ncbi.nlm.nih.gov/bioproject/PRJNA596510.

MESO-SCOPE (KM1709) environmental metadata are available at http://scope.soest.hawaii.edu/data/mesoscope/mesoscope.html.

HOE-Legacy 4 (KOK1607) environmental metadata are available at http://scope.soest.hawaii.edu/data/hoelegacy/documents.

Count values tables for metagenomes, and metatranscriptomes, as well as internal standard and volume conversion factor tables for use in the normalization of metatranscriptome raw counts, are available in the open public database Zenodo https://doi.org/10.5281/zenodo.15178202.

Supplementary text

Supplementary methods

Preliminary *Prochlorococcus* ASV ecotype differentiation and generation of phylogenetic trees

Using Amplicon Sequence Variants (ASVs) assigned by the DADA2 program, *Prochlorococcus* ecotype differentiation was achieved through the following methods. From the IMG genomes portal, *Prochlorococcus* and *Synechococcus* full-length 16S ribosomal gene reference sequences were downloaded[1]. Ecotype designations for isolated *Prochlorococcus* strains were cross-referenced with literature designations. All sequences were aligned using the MUSCLE alignment algorithm[2]. Finally, a maximum likelihood tree was built using 1000 bootstraps on the W-IQ-TREE web interface[3]. Tree visualization was done using the iTOL: Interactive Tree of Life online tool[4]. Bootstrap values of 70 and higher were reported.

Ribosomal RNA operon clone library generation

DNA was extracted from seawater samples obtained above and within the cyclone during the MESOSCOPE cruise. To produce amplicons spanning the ITS and 16S rRNA genes, PCR

amplification was performed using 27-F (5' AGRGTTYGATYMTGGCTCAG 3') and ACMITS-3-R (5'TCATCGCCTCTGTGTGCC 3') primers[5–7]. Amplicons were then cloned using a TOPO-TA cloning kit according to manufacturer's instructions (ThermoFisher K4575J10, Waltham MA). Competent cells were cultured on Lysogeny broth (LB)-medium with kanamycin. After growth, individual colonies were picked and sub-cultured on LB-medium with ampicillin. The sub-cultures were then picked and incubated in a liquid LB broth for three days before plasmid extraction with a QIAprep Spin Miniprep Kit (27104 Qiagen, Hilden Germany). Plasmids underwent PCR amplification using the M13 primers included in the TOPO-TA cloning kit. After amplification, the PCR product was sent to the Genewiz sequencing facility in South Plainfield, New Jersey, for Sanger sequence generation. To ensure the sequencing of the total ITS and 16S rRNA gene regions, internal primer pairs were used. A version of the forward and reverse complement was used for several primers. The following are the sequencing primers used to generate sequences: 27-F (5' AGRGTTYGATYMTGGCTCAG 3'), 515-F (5' GTGYCAGCMGCCGCGGTAA3'), 1492-F (5' AAGTCGTAACAAGGTARCCGTA 3'), 515-R (TTACCGCGGCKGCTGRCAC 3'), 1492-R (5' TACGGYTACCTTGTTACGACTT 3'), ACMITS-3-R (5'TCATCGCCTCTGTGTGCC 3'). Contigs were assembled using the Geneious assembler set using the highest sensitivity. Cloned sequences of 16S rRNA gene - ITS gene - partial 23S rRNA region from the MESO-SCOPE cruise are available at https://www.ncbi.nlm.nih.gov/bioproject/PRJNA596510.

Kruskal-Wallis and Wilcoxon rank sum tests

To test the significance of the enrichment of the single HLI-ASV in the cyclones of the HOE-Legacy 4 and MESO-SCOPE research cruises, both a Kruskal-Wallis and a Wilcoxan rank sum test were performed. Data was normalized by percentage. Using the 'Stats' package in base R, the functions Kruskal.test() and pairwise.wilcox.test() were used.

MAGs ID to Ecotype to GTDB Genus taxonomy mapping

ITS sequences annotated with ecotype designations were retrieved from the Integrated Microbial Genomes and Microbiomes *Prochlorococcus* portal[8] and from the Single-cell genome

database[9]. Representative genomes of the *Prochlorococcus* genera based on the NCBI taxonomy were retrieved from the Genome Taxonomy Database[10] v202. Primer sequences (ITS-F: 5′-CCGAAGTCGTTACTYYAACCC-3′ and ITS-R 5′-TCATCGCCTCTGTGTGCC-3′) were used *in silico* (https://github.com/egonozer/in_silico_pcr) to extract ITS sequences from the GTDB genomes. A homology search was performed using Blastn to compare the ITS sequences extracted from the genomes against the ITS sequence database to acquire their putative ecotype designations. This was used as input to generate the alluvial plot (Supplementary Figure 4) using Rawgraph[11].

Plotting of sea level anomaly

Sea level anomaly (SLA) was obtained from Simons CMAP[12] using Python pycmap package (version 0.2.19). The function plot_map() was used with the following variables to retrieve data specific to the date and locations relevant to the MESO-SCOPE cruise (tables=['tblAltimetry_REP_Signal'], variables=['sla'],dt1='2017-07-04', dt2='2017-07-04', lat1=23, lat2=28, lon1=-160.3403, lon2=-156.2829, depth1=0, depth2=0.5, exportDataFlag=True, show=True, levels=3). Detailed methods describing eddy identification, tracking, and sampling are reported elsewhere[13].

Supplementary references

1. Nordberg, H. *et al.* The genome portal of the Department of Energy Joint Genome Institute: 2014 updates. *Nucleic Acids Res.* **42**, D26–D31 (2014).

2. Edgar, R. C. MUSCLE: multiple sequence alignment with high accuracy and high throughput. *Nucleic Acids Res.* **32**, 1792–1797 (2004).

3. Trifinopoulos, J., Nguyen, L.-T., von Haeseler, A. & Minh, B. Q. W-IQ-TREE: a fast online phylogenetic tool for maximum likelihood analysis. *Nucleic Acids Res.* **44**, W232–W235 (2016).

4. Letunic, I. & Bork, P. Interactive Tree Of Life (iTOL): an online tool for phylogenetic tree display and annotation. *Bioinformatics* **23**, 127–128 (2007).

5. Zinser, E. R. *et al. Prochlorococcus* Ecotype Abundances in the North Atlantic Ocean As Revealed by an Improved Quantitative PCR Method. *Appl. Environ. Microbiol.* **72**, 723–732 (2006).

6. Heuer, H., Krsek, M., Baker, P., Smalla, K. & Wellington, E. M. Analysis of actinomycete communities by specific amplification of genes encoding 16S rRNA and gel-electrophoretic separation in denaturing gradients. *Appl. Environ. Microbiol.* **63**, 3233–3241 (1997).

7. Singer, E. *et al.* High-resolution phylogenetic microbial community profiling. *ISME J.* **10**, 2020–2032 (2016).

8. Martiny, A. C., Tai, A. P. K., Veneziano, D., Primeau, F. & Chisholm, S. W. Taxonomic resolution, ecotypes and the biogeography of *Prochlorococcus*. *Environ. Microbiol.* **11**, 823–832 (2009).

9. Berube, P. M. *et al.* Single cell genomes of *Prochlorococcus*, Synechococcus, and sympatric microbes from diverse marine environments. *Sci. Data* **5**, 180154 (2018).

10. Parks, D. H. *et al.* A standardized bacterial taxonomy based on genome phylogeny substantially revises the tree of life. *Nat. Biotechnol.* **36**, 996–996 (2018).

11. RAWGraphs: A Visualisation Platform to Create Open Outputs. in *CHItaly ’17* (Cagliari, Italy). doi:10.1145/3125571.3125585.

12. Ashkezari, M. D. *et al.* Simons Collaborative Marine Atlas Project (Simons CMAP): An open-source portal to share, visualize, and analyze ocean data. *Limnol. Oceanogr. Methods* **19**, 488–496 (2021).

13. Barone, B. *et al.* Biogeochemical Dynamics in Adjacent Mesoscale Eddies of Opposite Polarity. *Glob. Biogeochem. Cycles* **36**, (2022).

Supplementary figures

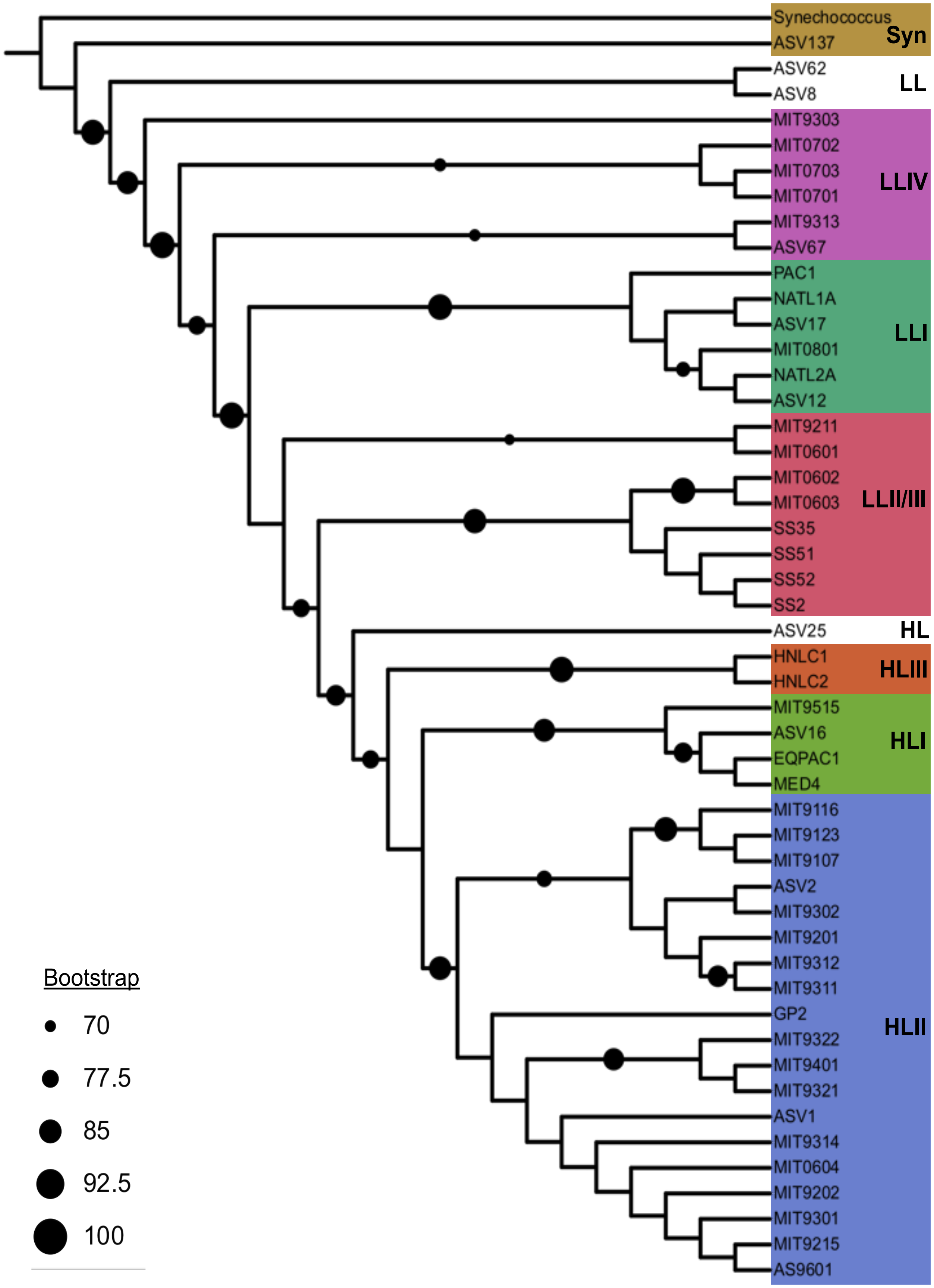

Supplementary Figure 1. **16S rRNA gene Maximum likelihood tree with sanger and amplicon sequences.** Phylogenetic tree showing the possible *Prochlorococcus* ecotype placement of abundant Amplicon Sequence Variants (ASVs) prevalent in both the MESO-SCOPE and HOE-Legacy 4 research cruises and full-length 16S rRNA gene Sanger-derived cloned sequences from specific pooled samples from the MESO-SCOPE cruise. Reference sequences are full-length 16S rRNA genes extracted from metagenome-assembled genomes of known ecotype affiliation in the Joint Genome Institute (JGI) database. The tree was constructed with 1000 bootstraps using the IQ-TREE web server. Bootstrap values are indicated by the size of the black dots.

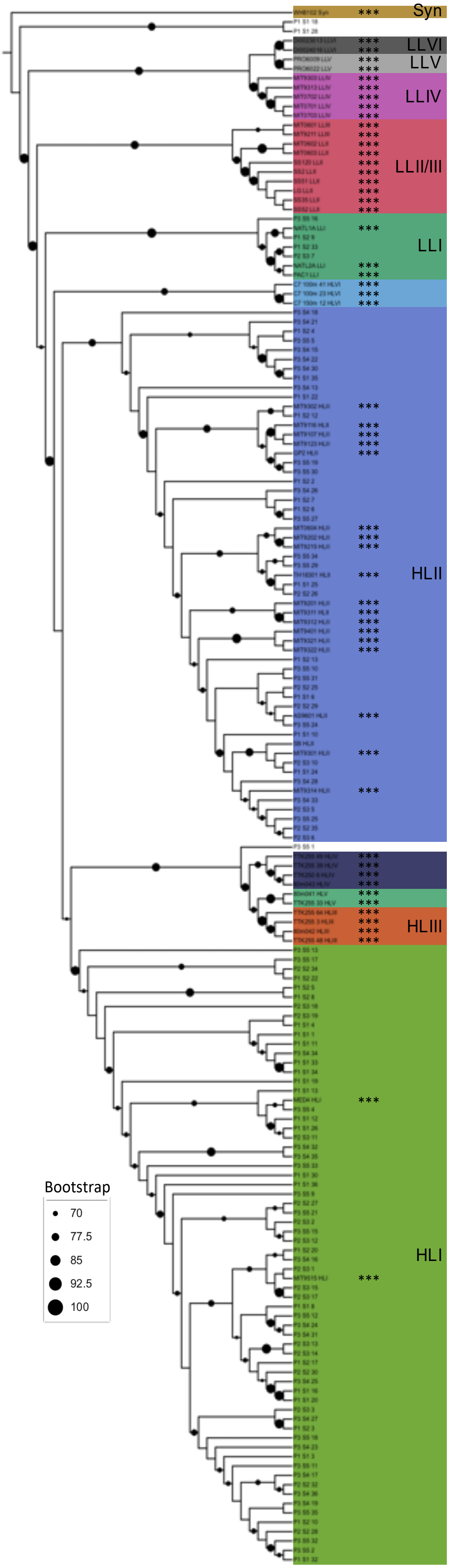

Supplementary Figure 2. **Maximum likelihood tree of *Prochlorococcus* ITS sequences from cloned 16S rRNA-ITS-23S rRNA PCR amplified regions from the MESOSCOPE expedition.** The dendrogram shows the phylogenetic placement of full-length ITS sequences from cloned 16S rRNA-ITS-23S rRNA PCR amplicons, from pooled depth profile samples collected during the MESO-SCOPE expedition. Reference sequences, indicated by three asterisks, represent full-length ITS sequences from *Prochlorococcus* isolates of known ecotype affiliation. The tree was constructed using 1000 bootstrap re-samplings with the IQ-TREE web server (Supplementary Figure 1).

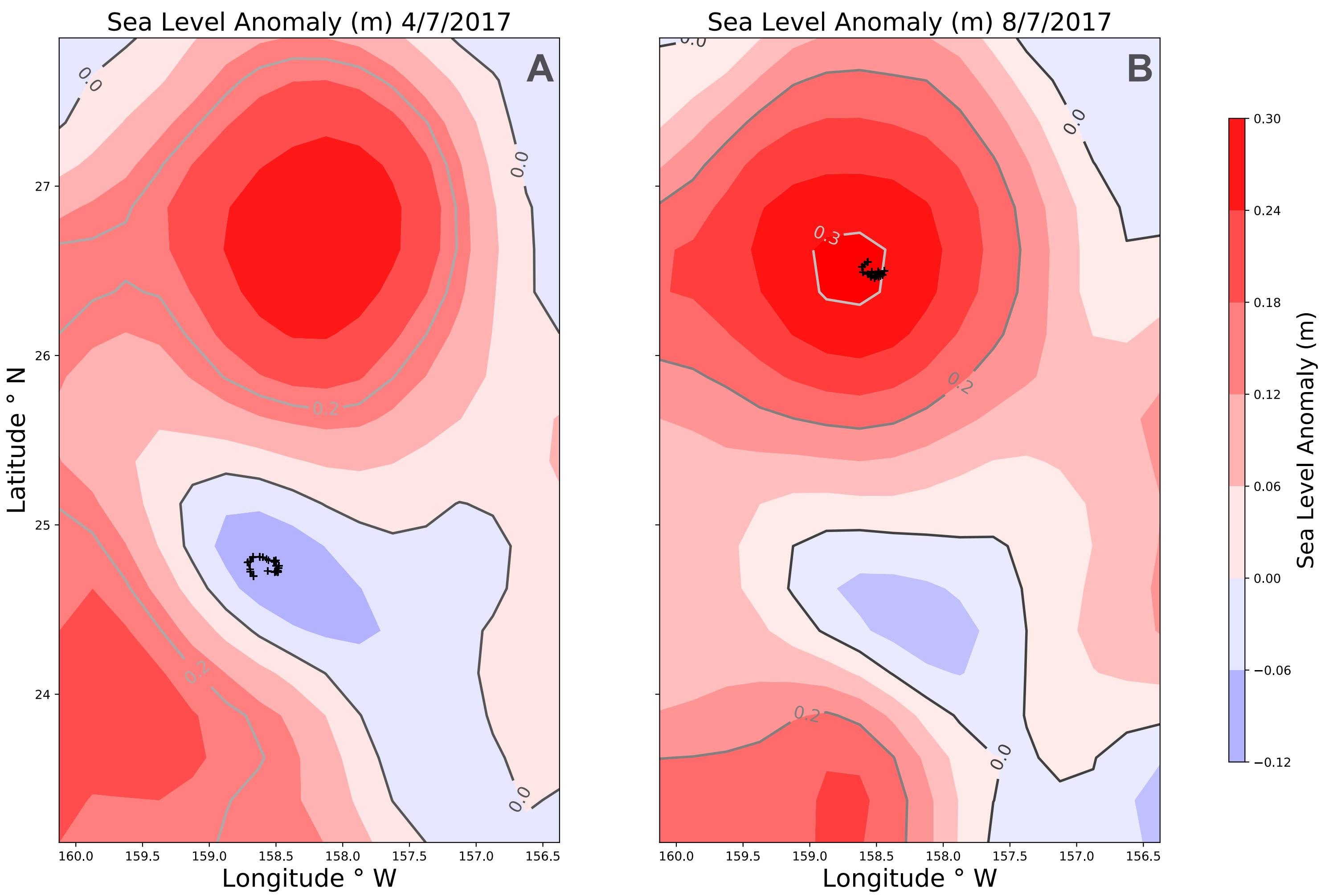

Supplementary Figure 3. **Eddies and Sampling Locations at Eddy Centers during the MESOSCOPE expedition.** Sea level anomaly (SLA) map displaying sampling positions superimposed on the SLA field from a representative day within the sampling period of each Lagrangian station: (a) Station 16 (or L1) at the cyclone center, and (b) Station 17 (or L2) at the anticyclone center. Positive SLA is depicted in red, while negative SLA is shown in blue.

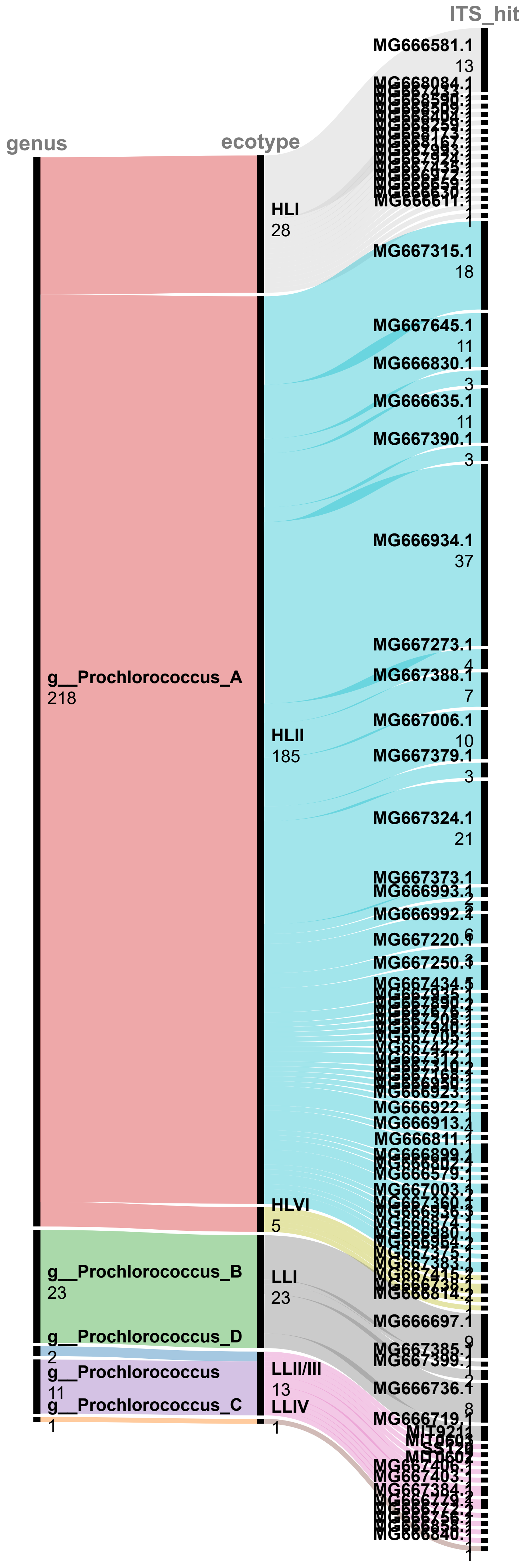

Supplementary Figure 4. **Mapping of ITS hit to ecotype to GTDB genera taxonomy.** Ecotype affiliation according to ITS sequences of different *Prochlorococcus* genomes and their GTDB genera taxonomy (Supplementary Table 4).

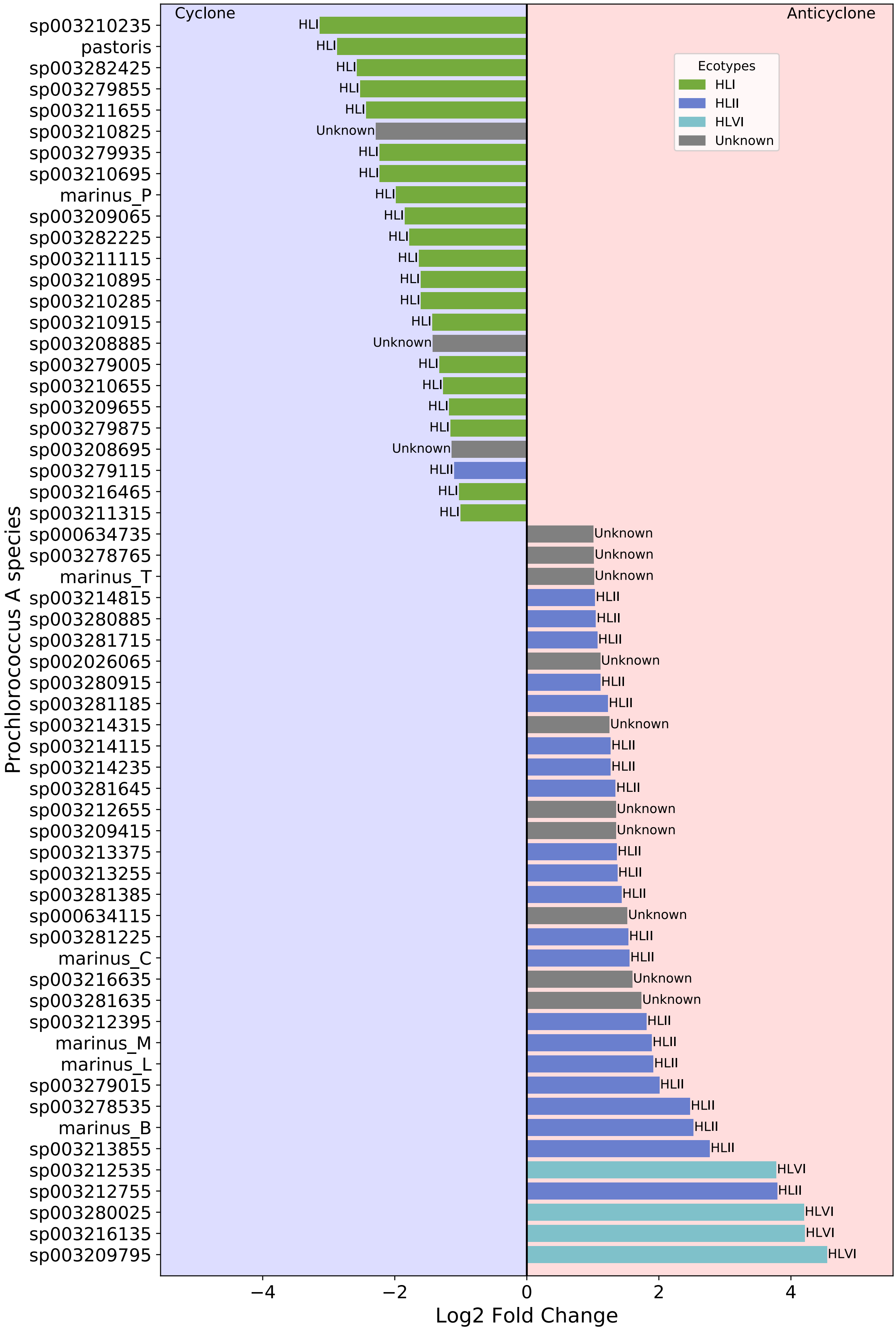

Supplementary Figure 5. **Overrepresentation of *Prochlorococcus* transcripts between adjacent eddies at the DCM sampled during the MESO-SCOPE cruise.** Transcript counts log2 fold change between the anticyclone and the cyclone of species-level annotation in the DCM. Fold change was calculated by dividing the mean of the species expression sum value of the anticyclone by the cyclone for each species (n=18 for each location). Only species with adjusted *P* values lower than 0.05 and a log2 fold change value higher than 2 for the anticyclone and smaller than -2 for the cyclone are shown. Statistical significance was calculated using the Kruskal-Wallis H-test. *P* values were corrected using the Benjamini-Hochberg procedure. Ecotype affiliation (adjacent to each bar head) according to genome to ITS mapping analysis is shown in Supplementary Figure 4.

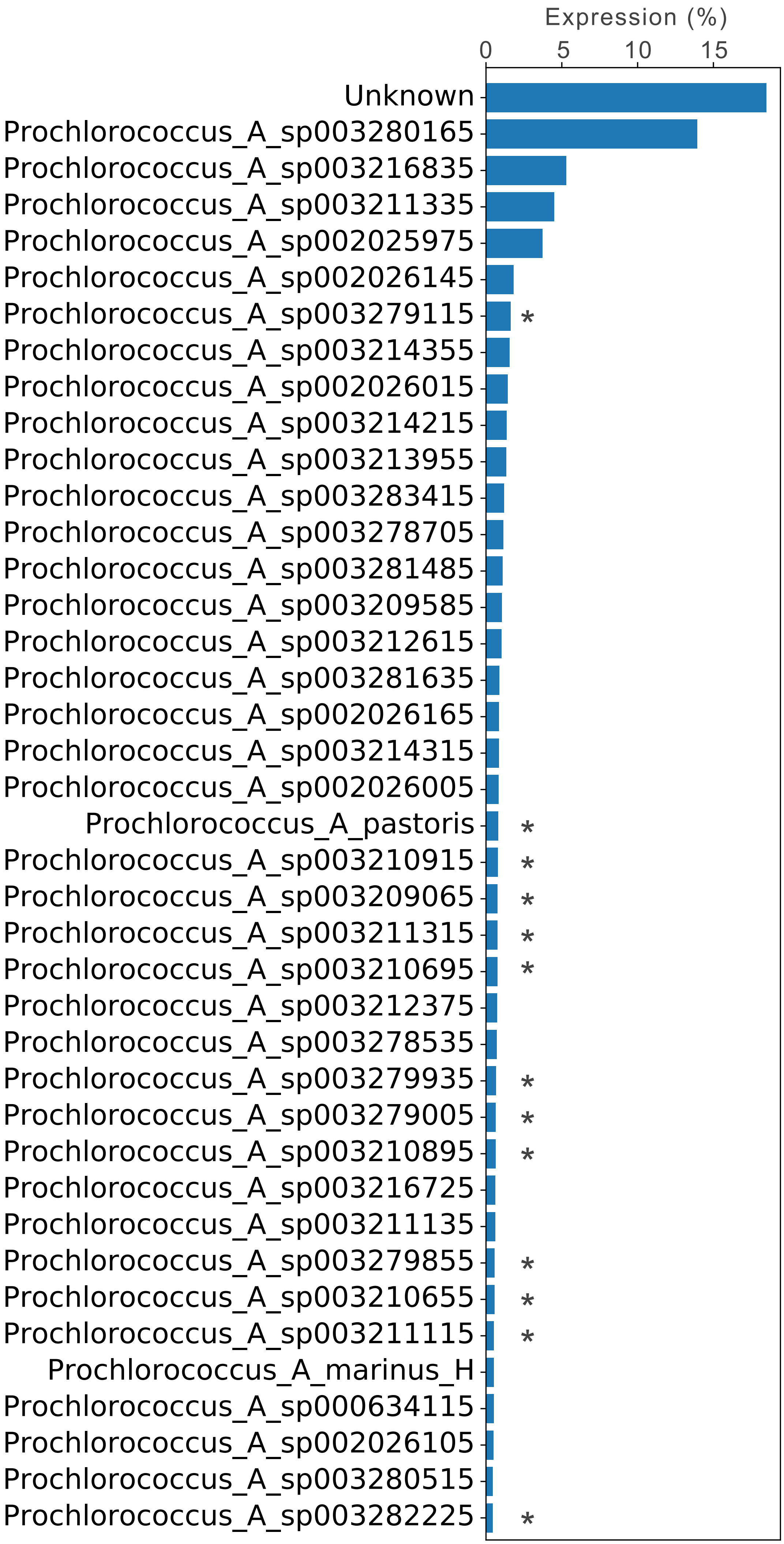

Supplementary Figure 6. **Percent expression of species within the *Prochlorococcus* A genus in the cyclone DCM.** Sum of species transcript read count as a percentage of *Prochlorococcus* A genus expression in the cyclone DCM samples. Designated by (*) are 13 species that were found to be significantly overrepresented in the cyclone vs the anticyclone DCM samples (Supplementary Figure 5).

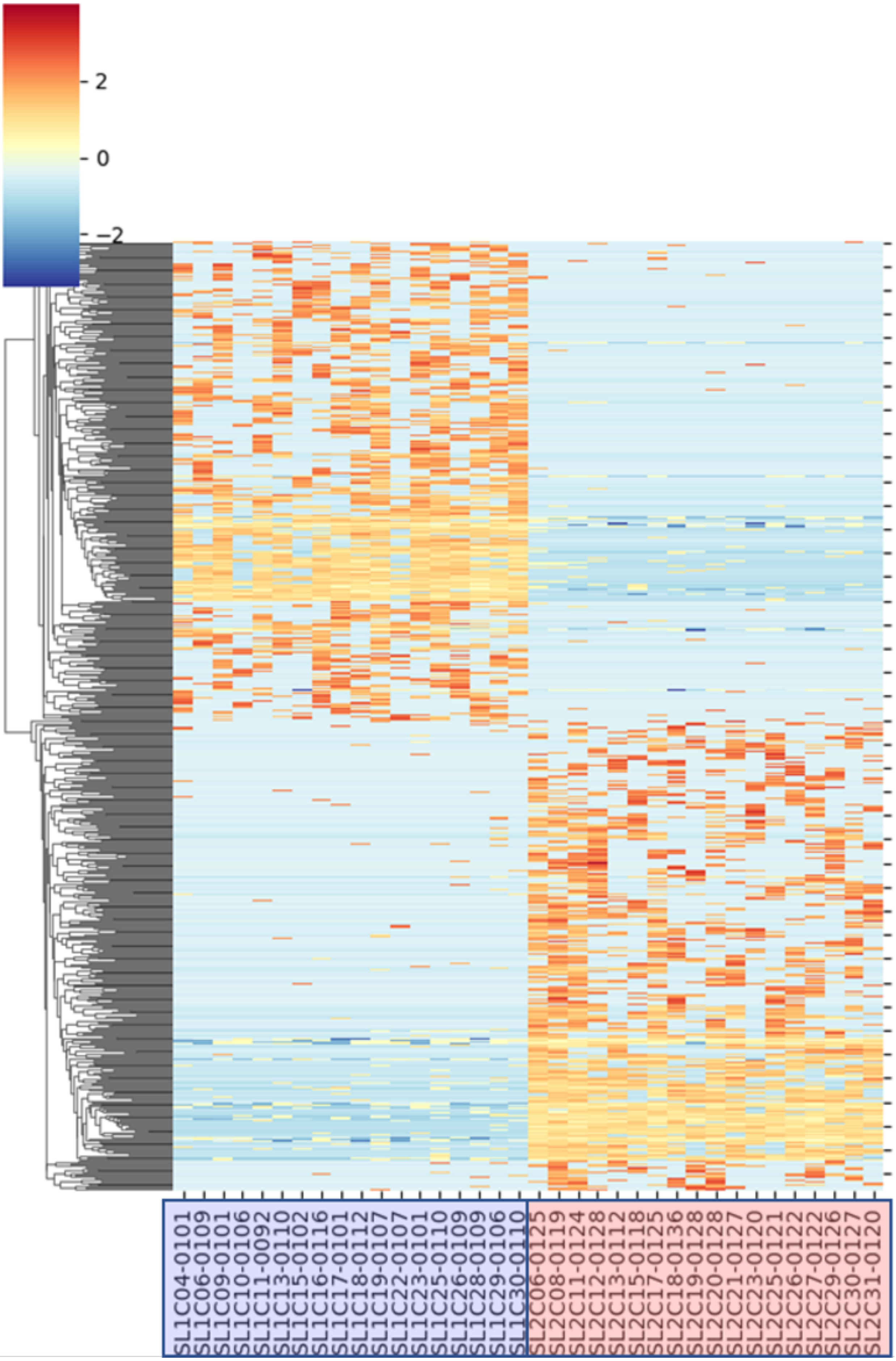

Supplementary Figure 7. **Differentially expressed transcripts between the eddies from the cyclone overrepresented *Prochlorococcus* A species.** Transcripts (rows, n=675) of the cyclone overrepresented species from the HLI ecotype as depicted in Supplementary Figure 5. Only significantly overexpressed transcripts (*P* values<0.05, n=18 in each location) are shown. Cyclone samples are highlighted in blue, and anticyclone samples in red (columns).

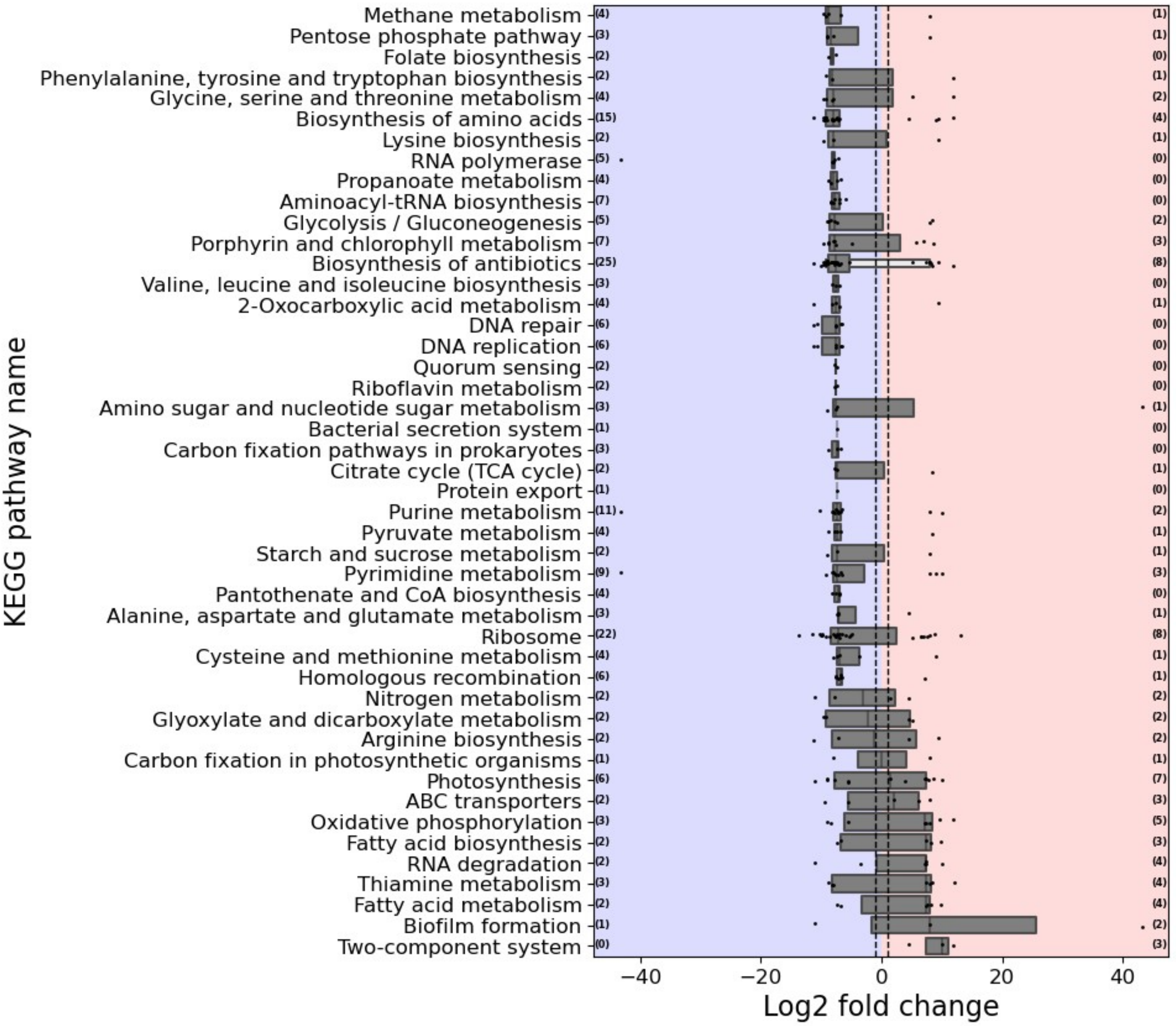

Supplementary Figure 8. **Transcription Fold Change Analysis of *Prochlorococcus* A species Transcripts in the DCM, Cyclone Vs. Anticyclone.** Box plot illustrating transcription log2 fold change data for *Prochlorococcus* A species HLI transcripts overrepresented in cyclone DCM, categorized by KEGG pathways. The number of transcripts in each category donated is in parentheses (Total of 213 transcripts). Each transcript can be plotted under multiple pathway categories. The red background highlights the positive log2 fold change for the anticyclone, while the blue background highlights the negative log2 fold change for the cyclone. The box represents the interquartile range (IQR) from Q1 to Q3, with the median indicated by a line within the box. Black dots denote data points for each transcript. The dashed lines mark the -2 and 2 log2 fold change values.

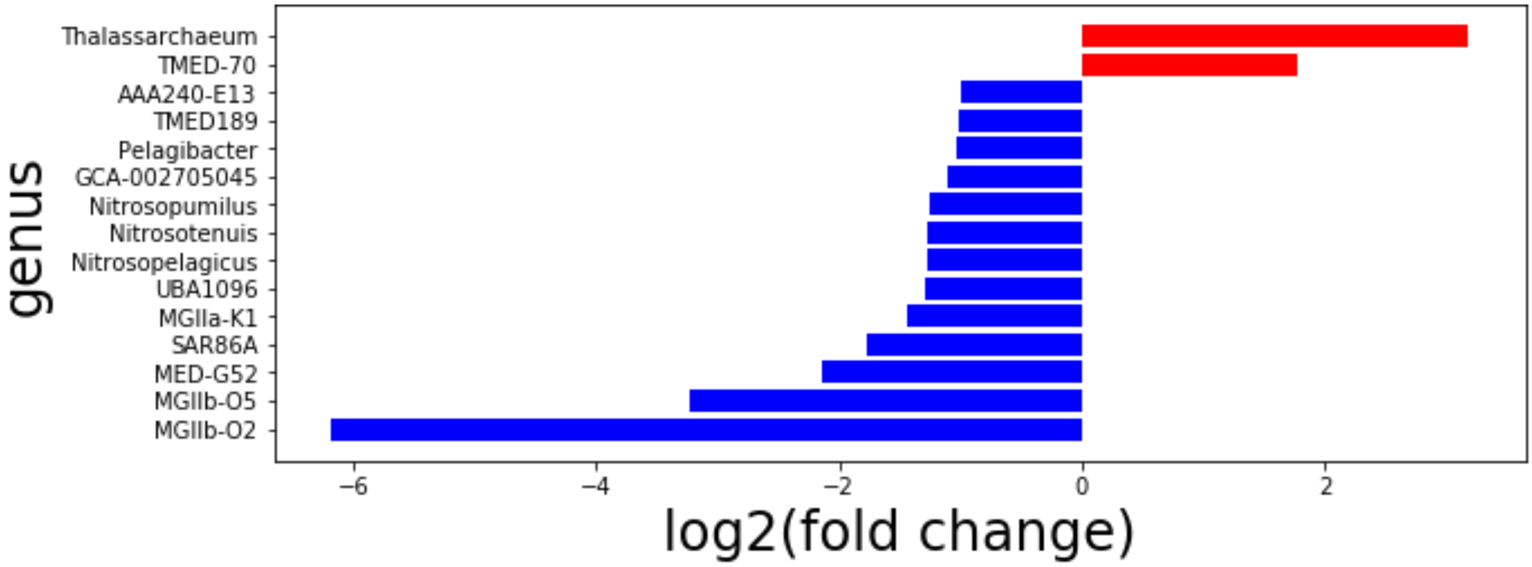

Supplementary Figure 9. **Overrepresented genera of heterotrophic prokaryotes between the eddies in the DCM depth sampled during the MESO-SCOPE cruise.** Transcript counts log2 fold change between the anticyclone and the cyclone of heterotrophic prokaryotes at the genus level annotation in the DCM. Fold change was calculated by dividing the mean expression sum value of the anticyclone by the cyclone for each genus (n=18 for each location). Only genera with adjusted *P* values lower than 0.05 and a fold change value higher than 2 for the anticyclone and smaller than ½ for the cyclone are plotted. Statistical significance was calculated using the Kruskal-Wallis H-test. *P* values were corrected using the Benjamini-Hochberg procedure.
